# A novel qutrit representation for RGB digital images

**DOI:** 10.1038/s41598-025-27910-0

**Published:** 2025-12-05

**Authors:** Mirna Rofail, Rasha Montaser, Ahmed Younes

**Affiliations:** 1 Department of Mathematics and Computer Science, Faculty of Science, Alexandria University, Alexandria, 21526 Egypt; 2 Department of Information Systems, Faculty of Computers and Information Science, Damanhour University, Damanhour, 22511 Egypt; 3 Faculty of Computer Science and Engineering, Alamein International University, Alamein, 51718 Egypt

**Keywords:** Ternary logic, Quantum image processing, Quantum ternary image circuit, Quantum image representation, Mathematics and computing, Physics

## Abstract

Ternary Quantum Image Processing (TQIP) leverages the power of qutrits to enhance Quantum Image Processing (QIP) by enabling higher information density, reducing error rates, and simplifying circuit complexity compared to traditional binary quantum systems. Inspired by the ENEQR binary model, this paper introduces the Ternary Novel Colored Quantum Representation (TNCQR), a qutrit-based model for encoding RGB digital images in a ternary quantum system. The TNCQR model requires fewer qutrits than the number of qubits needed in equivalent binary models. By incorporating a single ancilla qutrit, the model reduces the depth of high-complexity gates, thereby lowering the overall quantum cost and improving time complexity. To simplify the constructed circuits, an optimization algorithm is presented, consisting of 2 general phases applicable to any image size and one conditional phase specific to each image. This makes TNCQR an efficient, scalable, and resource-optimized quantum image representation model.

## Introduction

Quantum computing has proven its efficiency across multiple fields of computer science, from circuit design^1–3^ and algorithm development^4,5^ to advanced applications such as machine learning^6,7^, cryptography^8,9^, and quantum networking^10,11^. Among these, image processing plays a crucial role whether for analyzing visual datasets in quantum machine learning or for transmitting visual information through quantum communication channels. Therefore, the ability to efficiently represent digital images within quantum systems has become a fundamental prerequisite for the advancement of quantum information processing (QIP)^12–17^.

The field of quantum image representation (QIR) has evolved significantly over the past decade, with various models proposed to encode classical images in quantum states^12–17^. Early research focused on binary (qubit-based) models, such as the Novel Quantum Representation of Color Digital Images (NCQI)^18^, the Enhanced Quantum Representation based on the NEQR model (ENEQR)^14^, and the Mixed-State Representation of Quantum Color Images (MSR-QCI)^19^. These approaches encode position and color information using qubits, enabling various quantum image operations with time complexities $O(6qn2^{2n})$, $O(2n2^{2n})$, and $O(qn2^{2n})$ respectively. However, despite their contributions, these quantum models face several limitations such as growing the number of qubits rapidly with image size and color depth, high quantum cost, high time complexity and more storage space required to store quantum image especially for encoding and processing large-scale color images. These factors limit their scalability and practical feasibility on near-term quantum devices^20,21^.

To address these limitations, researchers have explored multi-valued quantum systems such as ternary (three-level quantum system). Ternary quantum systems require fewer physical resources and provide denser information encoding, enabling more compact circuit implementations compared to binary ones^22–25^. Ternary and hybrid representation models have been proposed to leverage these advantages, such as the Qutrit Representation of Quantum Images (QTRQ) model^15^, which introduces a ternary encoding for $3^n\times 3^n$ grayscale and RGB images with time complexity $O(qn3^{2n})$, and the Hybrid Qudit Digital Quantum Representation (HQDQR) model^16^, which combines qubits and qutrits to efficiently encode $2^n\times 3^m$ rectangle RGB images with time complexity $O((n+m)\times 2^n\times 3^m)$. While HQDQR demonstrates reduced quantum cost and better utilization of the Hilbert space through hybrid encoding, it is not a fully ternary scheme and still inherits certain binary limitations. Despite these advances, a fully ternary and lower-cost model for efficient RGB image representation remains unexplored, as existing models address only partial aspects of the problem.

To bridge these gaps, this paper introduces the Ternary Novel Colored Quantum Representation (TNCQR) model, a novel qutrit-based image representation model for encoding $3^n \times 3^n$ RGB digital images in ternary quantum systems. The proposed TNCQR leverages the advantages of qutrits to achieve more compact encoding, reduced quantum cost, and lower time complexity, while maintaining its scalability. The model is simulated and validated using the Cirq quantum simulator, as no commercial qutrit hardware currently exists^26–28^. Comparative analyses demonstrate that TNCQR achieves better quantum cost and time complexity than ENEQR, QTRQ, and HQDQR models.

The rest of this paper is organized as follows: The basics of ternary quantum logic, including detailed descriptions of each ternary logic gate and their associated quantum costs, are presented in the Supplementary Material. Section “Basic definitions” provides the fundamental definitions required throughout the paper. Section “Related work” introduces the concept of quantum image representation and reviews previous techniques for representing RGB images in ternary quantum systems. Section “Proposed TNCQR model” presents the proposed TNCQR model for representing $3^n \times 3^n$ RGB digital images. Section “TNCQR optimization algorithm” describes the optimization algorithm used to simplify the constructed circuits. Section “Performance analysis” reports the evaluation results and comparative analysis with existing models. Finally, the main conclusions and future directions are discussed in Sect. “Conclusion”.

## Basic definitions

The following definitions describe the main keywords used in describing and evaluating the ternary quantum circuits.

### Definition 1

(**Ancilla Qutrit**) An *ancilla* is a temporary, auxiliary three-level quantum state (qutrit) initialized in a specific known state, typically the $|0\rangle$ state^29,30^.

### Definition 2

(**Gate Count**) The gate count of a quantum circuit is the total number of quantum gates used in implementing a quantum circuit^29^.

### Definition 3

(**Time Complexity**) The time complexity is the number of sequential layers of gates (non-parallelized operations) that must be executed to realize the circuit (circuit depth)^13,31,32^.

### Definition 4

(**Quantum Cost (***QC***)**) Quantum cost is a metric that assigns costs to gates according to the ternary quantum system architecture. Quantum cost evaluates complex (*N*-qutrit) gates by decomposing them into elementary ternary gates and summing their costs^17,33–36^, as shown in S2.4 in the Supplementary Material.

## Related works

### Quantum image representation overview

Quantum image representation (QIR) provides a framework model for encoding classical digital images into quantum states, enabling the application of quantum algorithms for image storage, transformation, and processing. The goal of QIR models is to efficiently represent both pixel position and color intensity information using quantum bits (qubits) or higher-dimensional quantum systems (qudits), while minimizing the number of required quantum resources such as gate count, time complexity, and quantum cost.

Early research in quantum image processing introduced foundational models based on qubits. The Flexible Representation of Quantum Images (FRQI)^12^ was among the first to encode both position and grayscale intensity into a normalized quantum state using amplitude and phase information, achieving a time complexity of $O(2^{4n})$. Although FRQI provides a compact representation, its reliance on amplitude encoding makes image measurement and reconstruction complex and less precise. To overcome this limitation, the Novel Enhanced Quantum Representation (NEQR)^13^ encoded pixel values directly in the computational basis, which improves measurement accuracy and simplifies digital image operations, achieving a time complexity of $O(2qn2^{2n})$.

Based on NEQR, the Novel Quantum Representation of Color Digital Images (NCQI)^18^ extended this concept to RGB color images , achieving a time complexity of $O(6qn2^{2n})$, while the Enhanced Novel Quantum Representation (ENEQR)^14^ improved the encoding efficiency and reduced the quantum cost and achieving time complexity $O(2n2^{2n})$. The Mixed-State Representation of Quantum Color Images (MSR-QCI)^19^ improved efficiency and robustness by encoding image information using mixed quantum states ($\rho$), providing partial noise resilience and better scalability compared to pure-state models, achieving a time complexity of $O(qn2^{2n})$. These binary-based models established the feasibility of quantum image encoding and provided the foundation for further advancements in Quantum Image Processing (QIP) such as encryption, watermarking, and compression^37,38^.

However, as image sizes and color depths increase, qubit-based encoding require an exponentially growing number of qubits, leading to high quantum cost, time complexity, and storage overhead^20,21^.

### Ternary quantum image representation models

To address these limitations, researchers have started to explore multi-valued quantum systems such as qutrits (three-level quantum units), which can encode more information per quantum state and enable more compact and resource-efficient circuit implementations^23–25^. Ternary and hybrid quantum representation models, such as QTRQ^15^ and HQDQR^16^, have been proposed for representing colored RGB images in ternary quantum systems. The basics of ternary quantum logic are provided in detail in the Supplementary Material.

#### QTRQ representation model

The ternary QTRQ model^15^ is the first to represent grayscale images in ternary quantum systems. It employs two entangled qutrit sequences to store both the grayscale and position information, effectively storing the entire image in the superposition of these qutrit sequences.

The QTRQ model requires $2n + q$ qutrits to store a $3^n \times 3^n$ grayscale image with a gray scale [$0-3^q$]. Here, $q=6$ qutrits are used to represent the gray scale value in the range from 0 to 255 in each pixel. The expression for storing a $3^n \times 3^n$ quantum image using QTRQ is given as follows,

1 $$\begin{aligned} |Img\rangle =\dfrac{1}{3^n}\sum _{Y=0}^{3^n-1}\sum _{X=0}^{3^n-1}|f(Y,X)\rangle |YX\rangle =\dfrac{1}{3^n}\sum _{Y=0}^{3^n-1}\sum _{X=0}^{3^n-1}\bigotimes _{l=0}^{q-1}|C^{^l}_{YX}\rangle |YX\rangle . \end{aligned}$$

**Fig. 1:**
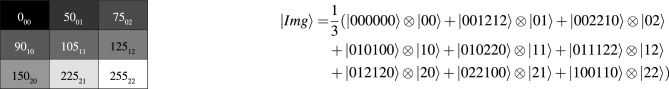
An example of a $3\times 3$ grayscale image with its representation expression using the QTRQ model.

The ternary sequence $C^{^0}_{YX} C^{^1}_{YX}... C^{^{q-1}}_{YX}$ encodes the gray value for a pixel *f*(*Y*, *X*) at position (*Y*, *X*), where $|C\rangle \in \{|0\rangle ,|1\rangle ,|2\rangle \}$. The time complexity for preparing a quantum image in the QTRQ model is $O(qn3^{2n})$ for a $3^n \times 3^n$ image. Figure 1 shows a $3 \times 3$ grayscale image and its corresponding quantum image state $|Img\rangle$.

Although QTRQ is primarily a ternary grayscale model, it can be extended to encode RGB digital images by adding 1 additional qutrit to the quantum circuit, where the 3 color channels (R, G, and B) are represented by the 3 qutrit states ($|0\rangle$, $|1\rangle$, and $|2\rangle$) using the ternary Hadamard (*H*) gate. The model requires $2n + q + 1$ qutrits to store a $3^n \times 3^n$ RGB image with a color range of [0, 255], where $q = 6$ qutrits are used to represent each color value for each pixel.

**Fig. 2:**
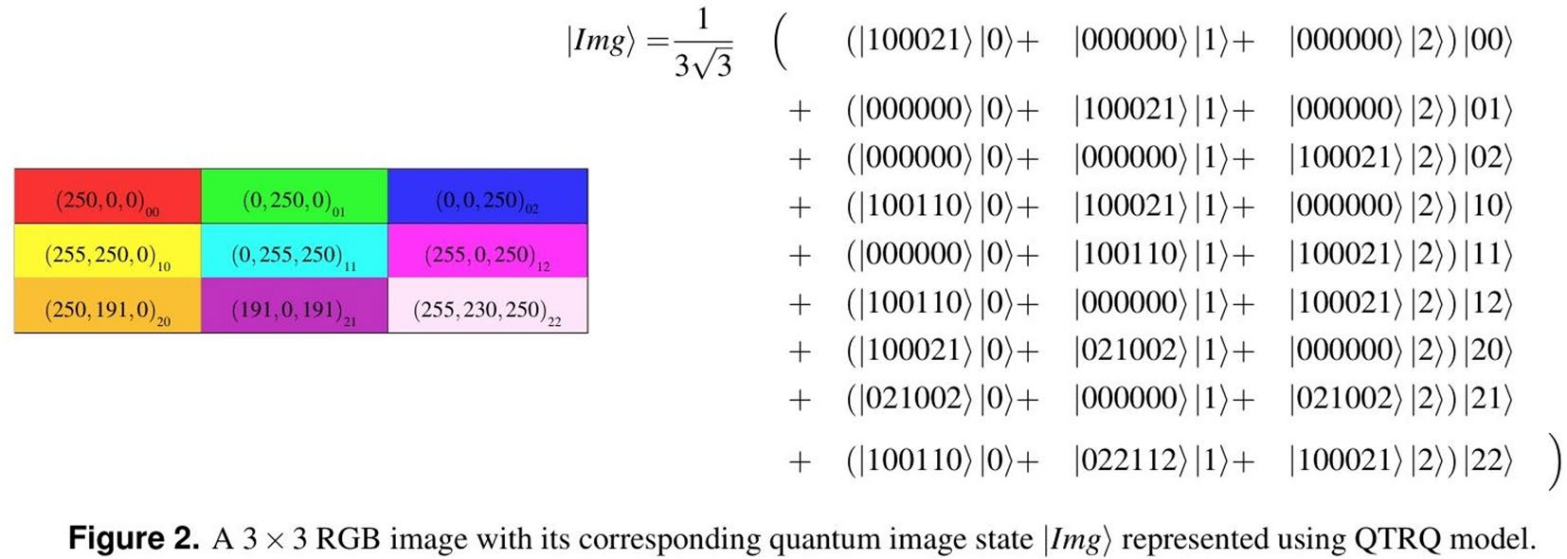
A $3\times 3$ RGB image with its corresponding quantum image state $|Img\rangle$ represented using QTRQ model.

**Fig. 3:**
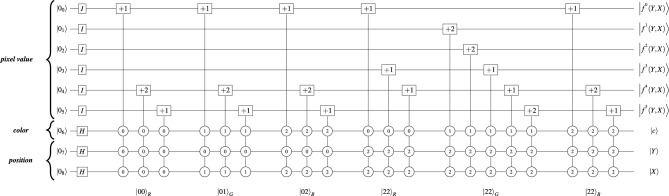
An example of encoding pixels (0, 0), (0, 1), (0, 2), and (2, 2) from the $3 \times 3$ RGB image in Fig. 2 using the QTRQ model, where $|f^{l}(Y,X)\rangle$ denotes the value of the $q_{_l}$ qutrit corresponding to the Red, Green, and Blue channels, with $l \in {0,1,\ldots ,q-1}$ .

Figure 2 present a $3\times 3$ RGB image and its corresponding quantum image state $|Img\rangle$ in the QTRQ model. Figure 3 shows the QTRQ implementation for pixels (0, 0), (0, 1), (0, 2), and (2, 2), demonstrating how the model encodes RGB digital images. The complete implementation of image in Fig. 2 results a ternary quantum circuit with a total cost $QC=637$.

Although the QTRQ model provides a compact ternary representation of quantum images using qutrits instead of qubits, it still faces several limitations. First, the model relies on a restricted set of ternary logic gates, leading to increased circuit depth and high time complexity $O(qn3^{2n})$. Second, the overall quantum cost remains high even for small-size images. Finally, as the image size increases ($3^n\times 3^n$), both the number of required qutrits and the circuit complexity grow rapidly, posing significant scalability challenges for large-scale or real-world images.

#### HQDQR representation model

**Fig. 4:**
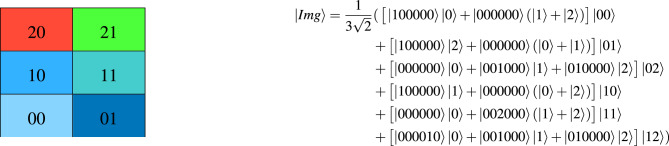
An example of a $3\times 2$ RGB image and its representation expression using the HQDQR model.

The Hybrid-qudit model (HQDQR)^16^ uses both qubits and qutrits to represent rectangular RGB images with a size of $2^n \times 3^m$. It efficiently encodes the color information of the RGB image using only 7 qutrits. For each pixel position, the model can use quantum registers composed of qubits, qutrits, or a combination of both, depending on which is most suitable. The expression for storing a rectangular RGB image using the HQDQR model is shown in Eq (2),

2 $$\begin{aligned} \begin{aligned} |Img\rangle&=\dfrac{1}{\sqrt{2^n3^{m+1}}}\sum _{Y=0}^{3^m}\sum _{X=0}^{2^n}\Omega _{_{XY}}(|000000\rangle \otimes (|0\rangle +|1\rangle +|2\rangle )\otimes |YX\rangle )\\&=\dfrac{1}{\sqrt{2^n3^{m+1}}}\sum _{Y=0}^{3^m}\sum _{X=0}^{2^n}(|R_{_{XY}}\rangle |0\rangle +|G_{_{XY}}\rangle |1\rangle +|B_{_{XY}}\rangle |2\rangle )\otimes |YX\rangle , \end{aligned} \end{aligned}$$

where $\Omega _{_{XY}}=\bigotimes _{_{l=0}}^{^5} \Omega ^{^{l,C}}_{_{XY}}, |C_{_{XY}}\rangle =|C^{^5}_{_{XY}} C^4_{_{XY}}... C^0_{_{XY}}\rangle , C=\{R,G,B\}$. Each $\Omega ^{^{l,C}}_{_{XY}}$ is a higher-order generalized hybrid Toffoli gate that flips the $l^{th}$ qubit of the color channel *C* at position $|YX\rangle$. Figure 4 shows a $3 \times 2$ RGB image and its corresponding quantum image state $|Img\rangle$. The time complexity for preparing a rectangular RGB quantum image is $O((m+n)2^n \times 3^m)$.

Although the Hybrid Qudit Digital Quantum Representation (HQDQR) model combines qubits and qutrits to encode RGB images in a mixed binary–ternary quantum system, offering improved resource utilization and better exploitation of the Hilbert space, it still presents several limitations. First, since it is a hybrid scheme, it is not a fully ternary representation and therefore inherits certain binary constraints, particularly in measurement and gate implementation. Second, the mixed qubit–qutrit structure increases circuit complexity and introduces synchronization challenges, which contribute to higher time complexity and circuit depth. Finally, although HQDQR demonstrates improved efficiency compared to earlier binary models, the paper does not provide a detailed quantitative analysis of quantum cost or gate-level optimization, making it difficult to fully evaluate its scalability for large-scale image encoding. To overcome these limitations, this paper proposed the TNCQR, a fully ternary model that leverages the advantages of qutrit-based computation to achieve a more compact, and resource-efficient encoding of $3^n\times 3^n$ RGB images in quantum systems.

## The proposed TNCQR model

This section introduces a Ternary Novel Colored Quantum Representation (TNCQR) model for representing colored RGB digital images in ternary quantum system inspired by the ENEQR binary quantum model^14^. The TNCQR model uses 7 qutrits to represent color information instead of 24 qubits in binary quantum system, indicating the efficiency of representing digital images in a ternary quantum system compared to a binary system.

To represent a $3^n \times 3^n$ RGB image using the TNCQR model, $q+2+2n$ qutrits are required, with a color scale $[0-3^q]$. Since the color scale ranges from 0 to 255, six qutrits ($q=6$) are required to store the color information for each pixel, as $3^6 > 256$. The $q+2+2n$ qutrits include an ancilla qutrit, this ancilla qutrit is used to load the position of a specific pixel to assign its color value. Each color (Red, Green, and Blue) is then individually loaded into the ancilla qutrit to set its value in the range of [0-255]. After setting the color values, the ancilla qutrit is reset to $|0\rangle$.

To prepare the quantum image using the proposed algorithm, assume that the initial state $|\Psi _0\rangle$ is a quantum register prepared with $q+2+2n$ qutrits all initialized by $|0\rangle$ to represent $3^n \times 3^n$ quantum image. The initial state $|\Psi _0\rangle$ is expressed as follows,

3 $$\begin{aligned} \begin{aligned} |\Psi _0\rangle =&\qquad |0\rangle ^{\otimes {q+2+2n}}\\ =&\underbrace{|000000\rangle }_{q \textit{ qutrits gives } 0-255} \otimes \underbrace{|0\rangle }_{\textit{ auxiliary qutrit }} \otimes \underbrace{|0\rangle }_{\textit{ color qutrit }} \otimes \underbrace{|0\rangle ^{\otimes 2n}}_{2n \textit{ position qutrits }}, \end{aligned} \end{aligned}$$

The 2*n* qutrits (last register) represent the $3^{2n}$ positions of the pixels of the image. The color qutrit (third register) is used to differentiate between the three colors (Red, Green, Blue) for each pixel. Each state in this color qutrit represents a color: state $|0\rangle$ for Red, state $|1\rangle$ for Green, and state $|2\rangle$ for Blue. Each pixel’s color value, ranging from 0 to 255, is represented using $q=6$ qutrits (first register) for each color. Finally, an ancilla qutrit (second register) is added to reduce the circuit cost by decreasing the number of *N*-qutrit gates used in the circuit, where $N \ge 3$. The entire image preparation process is divided into two steps:

***Step1 (S1):*** Prepare position and color information using two quantum operators $U_1, U_2$. The $U_1$ operator uses the single-qutrit gates *I* and *H* to prepare the position information for $3^{2n}$ pixels, as expressed in Eq (4),

4 $$\begin{aligned} U_1 = I^{\otimes q+2} \otimes H^{\otimes 2n}, \end{aligned}$$

It transforms the initial state $|\Psi _0\rangle$ into the intermediate state $|\Psi _1\rangle$ by applying 2*n* ternary *H* gates on the 2*n* position qutrits (last register in $|\Psi _0\rangle$), storing all pixels into the superposition of 2*n* qutrits, where $|YX\rangle$ represents a pixel at position (*Y*, *X*). The resulting state $|\Psi _1\rangle$ is the superposition of all pixels of a black image, as expressed in Eq (5),

5 $$\begin{aligned} \begin{aligned} |\Psi _1\rangle&= U_1 |\Psi _0\rangle \\&= (I|0\rangle )^{\otimes q+2} \otimes (H|0\rangle )^{\otimes 2n}\\&= \frac{1}{3^n} \sum ^{3^n-1}_{Y=0} \sum ^{3^n-1}_{X=0} \quad \underbrace{|000000\rangle }_{q \textit{ qutrits }} \quad \otimes \underbrace{|0\rangle }_{\textit{auxiliary qutrit}} \otimes \underbrace{|0\rangle }_{\textit{color qutrit}} \otimes \quad |YX\rangle . \end{aligned} \end{aligned}$$

The $U_2$ operator applies a single ternary *H* gate on the color qutrit (third register in $|\Psi _1\rangle$), to prepare the RGB color channels, as expressed in Eq (6),

6 $$\begin{aligned} U_2 = I^{\otimes q+1} \otimes H \otimes I^{\otimes 2n}, \end{aligned}$$

Applying the $U_2$ operator on the $|\Psi _1\rangle$ state results in the $|\Psi _2\rangle$ state, which indicates the RGB channels for each pixel, as expressed in the following Eq (7),

7 $$\begin{aligned} \begin{aligned} |\Psi _2\rangle&= U_2 |\Psi _1\rangle \\&= \frac{1}{3^n} \sum ^{3^n-1}_{Y=0} \sum ^{3^n-1}_{X=0} |000000\rangle \otimes |0\rangle \otimes H|0\rangle \otimes |YX\rangle \\&=\frac{1}{3^n\sqrt{3}} \sum ^{3^n-1}_{Y=0} \sum ^{3^n-1}_{X=0} |000000\rangle \otimes |0\rangle \otimes (|0\rangle _R+|1\rangle _G+|2\rangle _B) \otimes |YX\rangle \\&=\frac{1}{3^n\sqrt{3}} \sum ^{3^n-1}_{Y=0} \sum ^{3^n-1}_{X=0} |0\rangle ^{\otimes {q+1}} \otimes (|0\rangle _R+|1\rangle _G+|2\rangle _B) \otimes |YX\rangle . \end{aligned} \end{aligned}$$

***Step2 (S2):*** To complete the image preparation process, it is necessary to assign the RGB color value to each of the $3^{2n}$ pixels. This step is divided into $3^{2n}$ sub-operations to set the RGB color value for every pixel. For pixel ($Y_0$,$X_0$), the quantum sub-operation $U_{_{Y_0 X_0}}$ is expressed as in Eq (8),

8 $$\begin{aligned} U_{_{Y_0 X_0}} = \Bigl (I^{^{\otimes {q+2}}} \otimes \sum ^{3^n-1}_{v=0} \sum ^{3^n-1}_{\begin{array}{c} u=0\\ vu \ne Y_0 X_0 \end{array}} |vu\rangle \langle vu|\Bigr ) + \varpi _{_{{Y_0}{X_0}}} \otimes |Y_0 X_0\rangle \langle Y_0 X_0|, \end{aligned}$$

where $\varpi _{Y_0X_0}$ is the color-setting quantum operation that assigns the RGB color value to the specified pixel $(Y_0,X_0)$, and $|vu\rangle$ represents the other pixels in the image. The $\varpi _{_{Y_0X_0}}$ operation consists of several sub-operations $\omega ^1, \omega ^2_{_{cr}},$ and $\Omega _{_{cr}}$, as expressed in Eq (9), to assign the RGB color value to the pixel $(Y_0,X_0)$.

9 $$\begin{aligned} \varpi _{_{Y_0X_0}} = \Bigl (\omega ^{^1}_{_{Y_0X_0}}\Bigr ).\Bigl (\omega ^{^2}_{_R}\Bigr ).\Bigl (\Omega _{_{R}}\Bigr ). \Bigl (\omega ^{^2}_{_R}\Bigr ).\Bigl (\omega ^{^2}_{_G}\Bigr ).\Bigl (\Omega _{_{G}}\Bigr ).\Bigl (\omega ^{^2}_{_G}\Bigr ). \Bigl (\omega ^{^2}_{_B}\Bigr ).\Bigl (\Omega _{_{B}}\Bigr ). \Bigl (\omega ^{^2}_{_B}\Bigr ).\Bigl (\omega ^{^1}_{_{Y_0X_0}}\Bigr ), \end{aligned}$$

where $\omega ^{^1}_{_{Y_0X_0}}$ sub-operation detects the pixel position $(Y_0,X_0)$ from the superposition of 2*n* position qutrits in quantum circuit and loads it into the ancilla qutrit. The $\omega ^2_{_{cr}}$ sub-operation detects the color *cr* from the superposition of the color qutrit and loads it into the ancilla qutrit, where $cr\in \{R_{ed},G_{reen},B_{lue}\}$. After loading a position of a pixel $(Y_0,X_0)$ and one color *cr* into the ancilla qutrit, the $\Omega _{_{cr}}$ sub-operation assigns the value of the color *cr* in ternary representation over the *q* qutrits, where $\Omega _{_{cr}}\in \{\Omega _{_{R}},\Omega _{_{G}},\Omega _{_{B}}\}$. Therefore, the sub-operation $\Omega _{_{cr}}$ consists of *q* unitary operators as follows,

10 $$\begin{aligned} \Omega _{_{cr}}=\bigotimes ^{^{q-1}}_{_{l=0}}\Omega _{_{cr}}^{^{l}}, \end{aligned}$$

where the $\Omega _{_{cr}}^{^{l}}$ unitary operator is a single two-qutrit M-S gate that applied to one of the six *q* qutrits to change its state from $|0\rangle$ to $|1\rangle$ or $|2\rangle$ according to the *cr* value as follows,

11 $$\begin{aligned} \Omega _{_{cr}}^{^{l}}:|0\rangle \rightarrow |0\oplus C^{^{^l}}_{_{cr}}\rangle . \end{aligned}$$

Applying $\Omega _{_{cr}}$ operation on the *q* qutrits to set one *cr* value for the pixel ($Y_0,X_0$) is expressed as follows,

12 $$\begin{aligned} \begin{aligned} \Omega _{_{cr}} (|0\rangle ^{\otimes q})&= \Biggl (\bigotimes ^{^{q-1}}_{_{l=0}}\Omega _{_{cr}}^{^{l}}\Biggr ) (|0\rangle ^{\otimes q})\\&= \Big (\Omega _{_{cr}}^{^0}\otimes \Omega _{_{cr}}^{^1}\otimes ...\otimes \Omega _{_{cr}}^{^{q-1}}\Big )(|0\rangle |0\rangle ...|0\rangle )\\&= |0\oplus C_{_{cr}}^{^0}\rangle \otimes |0\oplus C_{_{cr}}^{^1}\rangle \otimes ... \otimes |0\oplus C_{_{cr}}^{^{q-1}}\rangle \\&= |C_{_{cr}}^{^0} C_{_{cr}}^{^1} \, ... \, C_{_{cr}}^{^{q-1}}\rangle \\&=|cr_{_{Y_0X_0}}\rangle , \end{aligned} \end{aligned}$$

where the state $|cr_{_{Y_0X_0}}\rangle$ represents the assigned *cr* value over the six *q* qutrits, resulting in one of the states $|R_{_{Y_0X_0}}\rangle$, $|G_{_{Y_0X_0}}\rangle$, or $|B_{_{Y_0X_0}}\rangle$ for the assigned colors Red, Green, or Blue for the pixel ($Y_0,X_0$). After setting the color *cr* value using $\Omega _{_{cr}}$, another $\omega ^{^2}_{_{_{cr}}}$ sub-operation is used to reset the ancilla qutrit back to the pixel position $(Y_0,X_0)$ to set the remaining color values for the same pixel. Finally, the ancilla qutrit is reset to $|0\rangle$ by $\omega ^{^1}_{_{Y_0X_0}}$, preparing it to load the next pixel in the image.

Each of the $\varpi _{Y_0X_0}$ sub-operations is applied in the ternary quantum circuit for the TNCQR model as follows: the sub-operation $\omega ^{^1}_{_{Y_0X_0}}$ is a generalized *N*-qutrit M-S gate with 2*n* control and 1 target qutrits, where *n* determines the size of $3^n\times 3^n$ image. The sub-operations $\omega ^{^2}_{_{R}}$, $\omega ^{^2}_{_{G}}$, and $\omega ^{^2}_{_{B}}$ are two-qutrit M-S gates with control qutrits set to $|0\rangle$, $|1\rangle$, and $|2\rangle$ for the Red, Green, and Blue colors, respectively. The sub-operation $\Omega _{_{cr}}$ which assigns one *cr* value over the *q* qutrits is implemented by a maximum of 5 two-qutrit M-S gates, each with a control set to $|2\rangle$ because each number from 0 to 255 in ternary number representation requires modifying up to 5 ternary digits. After applying the $U_{Y_0X_0}$ operation on the quantum state $|\Psi _2\rangle$, the state transforms into $|\Psi _3\rangle$ as shown in Eq (13),

13 $$\begin{aligned} \begin{aligned} |\Psi _3\rangle =&\,U_{_{Y_0X_0}} (|\Psi _2\rangle )\\ =&\,U_{_{Y_0X_0}}\Biggl (\frac{1}{3^n\sqrt{3}} \sum ^{3^n-1}_{v=0} \sum ^{3^n-1}_{u=0} |0\rangle ^{\otimes q+1} \otimes (|0\rangle _R+|1\rangle _G+|2\rangle _B)\otimes |vu\rangle \Biggr )\\ =&\frac{1}{3^n\sqrt{3}}\, U_{_{Y_0X_0}}\Biggl ( \sum ^{3^n-1}_{v=0} \sum ^{3^n-1}_{\begin{array}{c} u=0\\ \\ vu \ne Y_0 X_0 \end{array}} \big (|0\rangle ^{\otimes q+1} (|0\rangle _R+|1\rangle _G+|2\rangle _B) |vu\rangle + |0\rangle ^{\otimes q+1} (|0\rangle _R+|1\rangle _G+|2\rangle _B) |Y_0X_0\rangle \big )\Biggr )\\ =&\frac{1}{3^n\sqrt{3}}\,\Biggl ( \sum ^{3^n-1}_{v=0} \sum ^{3^n-1}_{\begin{array}{c} u=0\\ \\ vu \ne Y_0 X_0 \end{array}} \big (|0\rangle ^{\otimes q+1} (|0\rangle _R+|1\rangle _G+|2\rangle _B) |vu\rangle + \varpi _{_{Y_0X_0}} |0\rangle ^{\otimes q+1} (|0\rangle _R+|1\rangle _G+|2\rangle _B) |Y_0X_0\rangle \big )\Biggr )\\ =&\frac{1}{3^n\sqrt{3}}\,\Biggl ( \sum ^{3^n-1}_{v=0} \sum ^{3^n-1}_{\begin{array}{c} u=0\\ \\ vu \ne Y_0 X_0 \end{array}} \big (|0\rangle ^{\otimes q+1} (|0\rangle _R+|1\rangle _G+|2\rangle _B) |vu\rangle + \varpi _{_{Y_0X_0}} (|0\rangle ^{\otimes q}|0\rangle _R+|0\rangle ^{\otimes q}|1\rangle _G +|0\rangle ^{\otimes q}|2\rangle _B) |0\rangle |Y_0X_0\rangle \big )\Biggr )\\ =&\frac{1}{3^n\sqrt{3}}\,\Biggl ( \sum ^{3^n-1}_{v=0} \sum ^{3^n-1}_{\begin{array}{c} u=0\\ \\ vu \ne Y_0 X_0 \end{array}} \big (|0\rangle ^{\otimes q+1} (|0\rangle _R+|1\rangle _G+|2\rangle _B) |vu\rangle \\&+ (\Omega _{_{R}}|0\rangle ^{\otimes q}|0\rangle _R+\Omega _{_{G}}|0\rangle ^{\otimes q}|1\rangle _G+\Omega _{_{B}}|0\rangle ^{\otimes q}|2\rangle _B) |0\rangle |Y_0X_0\rangle \big )\Biggr )\\ =&\frac{1}{3^n\sqrt{3}}\Biggl (\sum ^{3^n-1}_{v=0} \sum ^{3^n-1}_{\begin{array}{c} u=0\\ \\ vu \ne Y_0 X_0 \end{array}} |0\rangle ^{\otimes q+1}(|0\rangle _R+|1\rangle _G+|2\rangle _B)|vu\rangle +\Big (|R_{Y_0X_0}\rangle |0\rangle +|G_{Y_0X_0}\rangle |1\rangle +|B_{Y_0X_0}\rangle |2\rangle \Big )|0\rangle |Y_0X_0\rangle \Biggr )\\ =&\frac{1}{3^n\sqrt{3}}\Biggl (\sum ^{3^n-1}_{v=0} \sum ^{3^n-1}_{\begin{array}{c} u=0\\ \\ vu \ne Y_0 X_0 \end{array}} |0\rangle ^{\otimes q+1}(|0\rangle _R+|1\rangle _G+|2\rangle _B)|vu\rangle +|f(Y_0,X_0)\rangle |0\rangle _{\textit{aux}}|Y_0X_0\rangle \Biggr ). \end{aligned} \end{aligned}$$

where the states $|R_{Y_0X_0}\rangle , |G_{Y_0X_0}\rangle , |B_{Y_0X_0}\rangle$ represent the assigned Red scale, Green scale, and Blue scale values in ternary representation over the six *q* qutrits, respectively. The state $|f(Y_0,X_0)\rangle$ represents the assigned RGB color value for the pixel ($Y_0,X_0$).

The sub-operation $U_{Y_0 X_0}$ assigns the RGB value for only one pixel of its corresponding position. Therefore, $U_3$ is the quantum operation that assigns the RGB values for all $3^{2n}$ image pixels. The $U_3$ operation consists of $3^{2n}$ sub-operations $U_{Y_sX_t}$ to assign the RGB values for the whole image as follows,

14 $$\begin{aligned} U_3=\prod _{s=0}^{3^n-1}\prod _{t=0}^{3^n-1} U_{Y_sX_t}. \end{aligned}$$

So, applying $U_3$ to the state $|\Psi _2\rangle$ results in the final state $|\Psi _4\rangle$, which is the quantum RGB image of the TNCQR model as shown in Eq (15). The state $|\Psi _3\rangle$ represents applying the $U_{Y_0 X_0}$ operation to one pixel to assign its value, while $|\Psi _4\rangle$ represents applying the $U_3$ operation which assigns the $U_{Y_s X_t}$ to all $3^{2n}$ pixels.

15 $$\begin{aligned} \begin{aligned} |\Psi _4\rangle&=U_3|\Psi _2\rangle \\&=\frac{1}{3^n\sqrt{3}}\sum _{Y=0}^{3^n-1}\sum _{X=0}^{3^n-1} \varpi _{YX}|0\rangle ^{\otimes q+1}(|0\rangle _R+|1\rangle _G+|2\rangle _B)|YX\rangle \\&=\frac{1}{3^n\sqrt{3}}\sum _{Y=0}^{3^n-1}\sum _{X=0}^{3^n-1} \Big (|R_{_{YX}}\rangle |0\rangle +|G_{_{YX}}\rangle |1\rangle +|B_{_{YX}}\rangle |2\rangle \Big )|0\rangle _{\textit{aux}}|YX\rangle \\&=\frac{1}{3^n\sqrt{3}}\sum _{Y=0}^{3^n-1}\sum _{X=0}^{3^n-1} |f(Y,X)\rangle |0\rangle _{\textit{aux}}|YX\rangle .\\ \end{aligned} \end{aligned}$$

After the two Steps ***S1*** and ***S2*** described above, the entire preparation of $3^n\times 3^n$ RGB image using the TNCQR model is done with time complexity $O(n3^{2n})$ as discussed in the following Lemma 1, Lemma 2, Lemma 3 and Theorem 1,

### Lemma 1

The time complexity of preparing pixel positions and color channels in S1 is *O*(1).

### Proof

Since $U_1$ (preparing the $3^{2n}$ pixel positions) and $U_2$ (preparing the three color channels) apply the $2n+1$ ternary Hadamard gates on $2n+1$ independent qutrits within a single, parallel layer, the circuit depth contributed by these operations is constant. Therefore, under the parallel-execution assumption, the time complexity (circuit depth) of both preparation stages is *O*(1). $\square$

### Lemma 2

The time complexity of the quantum operation $U_{Y_sX_t}$ in S2, which sets the RGB value for one pixel $(Y_s,X_t)$ is *O*(*n*).

### Proof

The main sub-operation in $U_{Y_sX_t}$ is $\varpi _{{Y_sX_t}}$, which is decomposed into several sub-operations. It begins with $\omega ^{^1}_{_{Y_sX_t}}$, which is implemented by a generalized *N*-qutrit M-S gate with 2*n* control qutrits and 1 target qutrit ($2n+1$-qutrit M-S gate) to load the pixel position into the ancilla qutrit. Then, the operations $\omega ^{^2}_{_R}$, $\omega ^{^2}_{_G}$, and $\omega ^{^2}_{_B}$, each consisting of a maximum of 7 two-qutrit M-S gates: 5 two-qutrit gates to set each color value in range [0-255] on $q=6$ qutrits, and 2 two-qutrit gates to load and unload each color channel on the ancilla qutrit. Finally, the ancilla qutrit is reset to $|0\rangle$. Therefore, for every $U_{Y_sX_t}$ operation, no more than two generalized *N*-qutrit gate + 21 two-qutrit gates are needed.

Each generalized $2n+1$-qutrit M-S gate can be decomposed into $8n-3$ two-qutrit M-S gates with the presence of $2n-1$ ancilla qutrits and 2*k* one-qutrit gates, where *k* is the number of controls not in state $|2\rangle$ and $k \in \{0,1,...,2n\}$. So, the time complexity of every $2n+1$-qutrit M-S gate is no more than *O*(*n*). So, the time complexity of the sub-operation $U_{Y_sX_t}$ is *O*(*n*) with the assistance of enough ancilla qutrits. $\square$

### Lemma 3

The time complexity of step S2, which sets the RGB value for all $3^{2n}$ pixels is $O(n3^{2n})$.

### Proof

The S2 step is divided into $3^{2n}$ sub-operations $U_{Y_sX_t}$ to store the RGB scale values for all $3^{2n}$ pixels. According to Lemma 2, the time complexity of the $U_{Y_sX_t}$ operation is *O*(*n*). For the main operation $U_3$, it applies $3^{2n}$ sub-operations which is the total number of pixels in a $3^n \times 3^n$ RGB image. Therefore, the time complexity of $U_3$ is $O(n3^{2n})$. $\square$

### Theorem 1

The time complexity of preparing $3^n\times 3^n$ RGB quantum image using the TNCQR model in ternary quantum system is $O(n3^{2n})$.

### Proof

The time complexity of quantum image preparation in the TNCQR model is calculated based on the quantum operations used in the previous steps S1 and S2. The operations used in S1 are $U_1$ and $U_2$, which prepare pixel positions and color channels. The time complexity of S1 is *O*(1), as proved in Lemma 1. S2 is divided into $3^{2n}$ sub-operations to assign the color value for each pixel. Since the main operation in this step is $U_{Y_sX_t}$ with time complexity *O*(*n*), the time complexity for S2 is $O(n3^{2n})$, as proved in Lemmas 2 and 3. Therefore, the time complexity of representing a $3^n \times 3^n$ RGB image in the TNCQR model is $O(1) + O(n3^{2n})$, which results $O(n3^{2n})$. $\square$

### TNCQR RGB image implementation

**Fig. 5:**
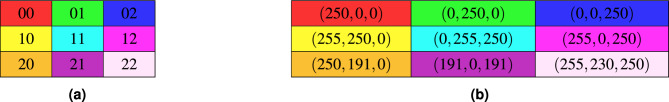
A $3 \times 3$ RGB image: (**a**) Represents the position of each pixel as index of *YX*. (**b**) The color values for each pixel in Red, Green, and Blue each in range $[0 - 255]$.

In this section, an RGB image is represented using the TNCQR model. Given a $3\times 3$ RGB image in Fig. 5, where Fig. 5a shows the 9 pixels positions and Fig. 5b shows the (R,G,B) color values for each pixel.

To represent the 9 pixels for a $3 \times 3$ RGB image, 10 qutrits are required as follows: using the formula $q+2+2n$, set the value of *n* to 1. This results in $2\times 1=2$ qutrits to represent the 9 pixels positions ($3^2=9$). Next, set $q=6$ because its the required number of qutrits to represent the decimal number from 0 to 255 as 6 digits in ternary representation, which represents each color value. Additionally, 1 qutrit is used to differentiate between the colors, and 1 ancilla qutrit is included, resulting in a total 10 qutrits.

To implement the quantum circuit representing the proposed model for a colored image with a size of $3 \times 3$, first, apply 3*H* gates to the 3 qutrits representing the pixel positions and the color qutrit. Then, use two-qutrit and three-qutrit gates to set the RGB color value for each pixel as follows:

1. A three-qutrit gate is used to load a specific pixel position onto the ancilla qutrit with two control qutrits representing the pixel position and $[+1]$ gate on the target qutrit (open position $\omega ^{^1}_{_{Y_0X_0}}$).
2. A two-qutrit gate with a control qutrit set to $|0\rangle$ and $[+1]$ gate on the target used to load the Red color onto the ancilla qutrit (load Red $\omega ^{^2}_{_R}$).
3. A maximum of 5 two-qutrit gates with control qutrit set to $|2\rangle$ and $[+1]$ or $[+2]$ gates on the target are used to assign the Red color value on the six *q* qutrit ($\Omega _{_{R}}$).
4. the Red color is unloaded from the ancilla qutrit by a two-qutrit gate with a control qutrit set to $|0\rangle$ and $[+2]$ gate on the target (unload Red $\omega ^{^2}_{_R}$).
5. Steps 2-4 are repeated to set the Green and Blue color values. The control qutrit of two-qutrit gate $\omega ^{^2}_{_G}$ is set to $|1\rangle$ for Green and to $|2\rangle$ in $\omega ^{^2}_{_B}$ for Blue.
6. The pixel position is closed by a three-qutrit gate with two control qutrits representing the position and $[+2]$ gate on the target (close position $\omega ^{^1}_{_{Y_0X_0}}$).
7. Steps 1-6 are repeated to represent each pixel in the image.

Every open gate has target $[+1]$ operation, and every close gate has target $[+2]$ operation. These $[+1]$ and $[+2]$ operations are inverse operations, as mentions in Sect. “S2.1” in the Supplementary Material.

The state $|\Psi _4\rangle$ in the expression shown in Eq. (16) represents the $3 \times 3$ RGB image in Fig. 5 using the TNCQR model. It shows the RGB values as decimal numbers from 0 to 255, then it represents the RGB values as 6 digits in the ternary number format.

16 $$\begin{aligned} \begin{aligned} |\Psi _3\rangle =&\frac{1}{3\sqrt{3}}\Bigl (\big (|250\rangle |0\rangle +|0\rangle |1\rangle +\quad|0\rangle |2\rangle \big )\quad |0\rangle _{aux}|00\rangle \\ \quad&+\quad\big (\quad|0\rangle |0\rangle +|250\rangle |1\rangle +\quad|0\rangle |2\rangle \big )\quad |0\rangle _{aux}|01\rangle \\ \quad&+\quad\big (\quad|0\rangle |0\rangle +\quad|0\rangle |1\rangle +|250\rangle |2\rangle \big )\quad |0\rangle _{aux}|02\rangle \\ \quad&+\quad\big (|255\rangle |0\rangle +|250\rangle |1\rangle +\quad|0\rangle |2\rangle \big )\quad |0\rangle _{aux}|10\rangle \\ \quad&+\quad\big (\quad|0\rangle |0\rangle +|255\rangle |1\rangle +|250\rangle |2\rangle \big )\quad |0\rangle _{aux}|11\rangle \\ \quad&+\quad\big (|255\rangle |0\rangle +\quad|0\rangle |1\rangle +|250\rangle |2\rangle \big )\quad |0\rangle _{aux}|12\rangle \\ \quad&+\quad\big (|250\rangle |0\rangle +|191\rangle |1\rangle +\quad|0\rangle |2\rangle \big )\quad |0\rangle _{aux}|20\rangle \\ \quad&+\quad\big (|191\rangle |0\rangle + \quad |0\rangle |1\rangle +|191\rangle |2\rangle \big )\quad |0\rangle _{aux}|21\rangle \\ \quad&+\quad\big (|255\rangle |0\rangle +|230\rangle |1\rangle +|250\rangle |2\rangle \big )\quad |0\rangle _{aux}|22\rangle \Bigr )\\ =&\frac{1}{3\sqrt{3}}\Bigl ((|100021\rangle |0\rangle +|000000\rangle |1\rangle +|000000\rangle |2\rangle )\quad |0\rangle _{aux}|00\rangle \\ \quad&+\quad(|000000\rangle |0\rangle +|100021\rangle |1\rangle +|000000\rangle |2\rangle )\quad |0\rangle _{aux}|01\rangle \\ \quad&+\quad(|000000\rangle |0\rangle +|000000\rangle |1\rangle +|100021\rangle |2\rangle )\quad |0\rangle _{aux}|02\rangle \\ \quad&+\quad(|100110\rangle |0\rangle +|100021\rangle |1\rangle +|000000\rangle |2\rangle )\quad |0\rangle _{aux}|10\rangle \\ \quad&+\quad(|000000\rangle |0\rangle +|100110\rangle |1\rangle +|100021\rangle |2\rangle )\quad |0\rangle _{aux}|11\rangle \\ \quad&+\quad(|100110\rangle |0\rangle +|000000\rangle |1\rangle +|100021\rangle |2\rangle )\quad |0\rangle _{aux}|12\rangle \\ \quad&+\quad(|100021\rangle |0\rangle +|021002\rangle |1\rangle +|000000\rangle |2\rangle )\quad |0\rangle _{aux}|20\rangle \\ \quad&+\quad(|021002\rangle |0\rangle +|000000\rangle |1\rangle +|021002\rangle |2\rangle )\quad |0\rangle _{aux}|21\rangle \\ \quad&+\quad(|100110\rangle |0\rangle +|022112\rangle |1\rangle +|100021\rangle |2\rangle )\quad |0\rangle _{aux}|22\rangle \Bigr ) \end{aligned} \end{aligned}$$

**Fig. 6:**
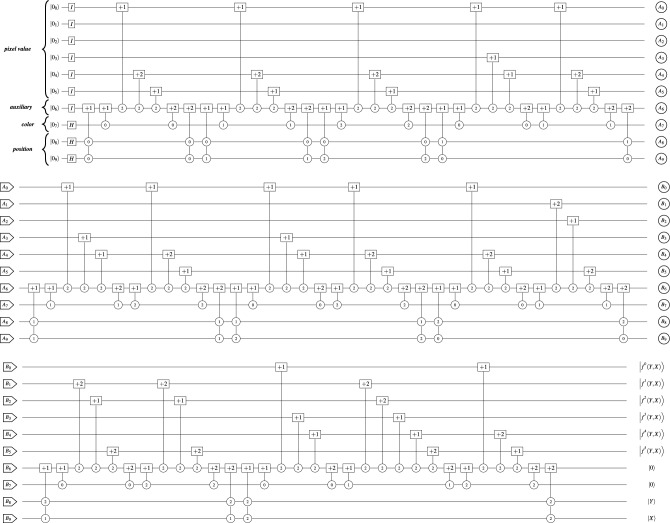
The quantum circuit for the TNCQR representation of the $3\times 3$ RGB image shown in Fig. 5, with $QC = 267$.

Figure 6 shows the quantum circuit that represents the implementation of the $3\times 3$ RGB image shown in Fig. 5 using the TNCQR model with 10 qutrits and $QC = 267$.

## TNCQR optimization algorithm

This section presents the optimization process in order to reduce the quantum cost of the constructed circuits from the TNCQR model, such as the circuit shown in Fig. 6. These steps are generic and applicable to images of any size. The optimization is divided into 3 phases, as shown in Algorithm 1.

**Algorithm 1:**
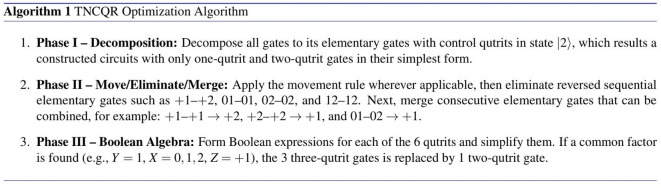
TNCQR Optimization Algorithm

### Phase I

In this phase, each *N*-qutrit gate ($N \ge 2$) is decomposed into its elementary gates in the simplest possible form. For a two-qutrit gate, the control qutrit must be in state $|2\rangle$ to achieve minimal complexity. Consequently, any two-qutrit gate whose control qutrit is in the $|0\rangle$ or $|1\rangle$ state is decomposed into 2 one-qutrit gates and 1 two-qutrit gate with a $|2\rangle$ control state, as illustrated in Fig. S2 of the Supplementary Material.

**Fig. 7:**
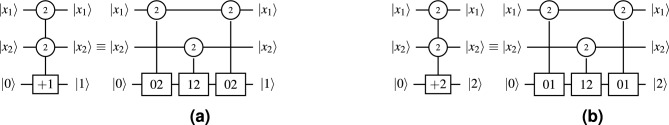
Decomposition of the ternary Toffoli gate as proposed in^17^ with a total quantum cost of $QC = 3$. (**a**) Decomposition for the $Z = +1$ gate. (**b**) Decomposition for the $Z = +2$ gate.

For ternary Toffoli gates, each gate is decomposed into its elementary gates as shown in Fig. S5 of the Supplementary Material. In this decomposition, each ternary Toffoli gate consists of five two-qutrit gates. However, Monfared^17^ proposed an alternative decomposition that reduces the gate count to 3 two-qutrit gates under specific conditions: both control qutrits must be in state $|2\rangle$ and the target qutrit must be initialized to $|0\rangle$, as shown in Fig. 7.

**Fig. 8:**
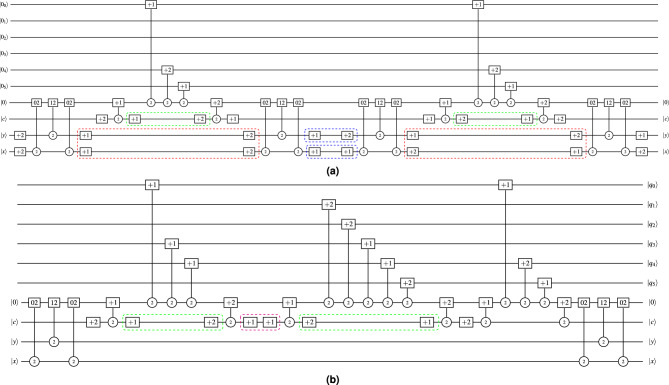
An example of phase I gate decomposition for pixels (0, 0), (0, 1) and (2, 2). (**a**) Decomposition of pixels (0, 0) and (0, 1) (**b**) Decomposition of pixel (2, 2).

To apply this phase to the TNCQR circuit shown in Fig. 6, Fig. 8 presents an example of decomposing the gates for pixels (0, 0), (0, 1), and (2, 2), where the two-qutrit gates that represent the pixel positions and the one-qutrit gates that represent the Red and Green colors are decomposed. For every open position gate labeled $+1$, there is a corresponding closed gate labeled $+2$. After decomposition, the 02, 12, and 22 gates become self-inverse, as explained in Sect. “S2.1” of the Supplementary Material. The resulting circuit of Phase I has a total quantum cost of $QC = 231$.

### Phase II

**Fig. 9:**
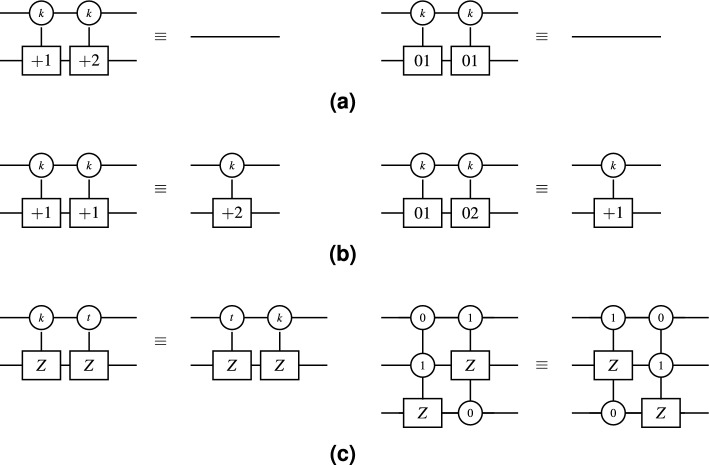
Examples of optimization rules described in^15^. (**a**) Elimination Rule examples, where $k \in \{0,1,2\}$. (**b**) Merger Rule examples, where $k\in \{0,1,2\}$. (**c**) Movement Rule example, where $k \ne t$ and $k,t \in \{0,1,2\}$.

**Fig. 10:**
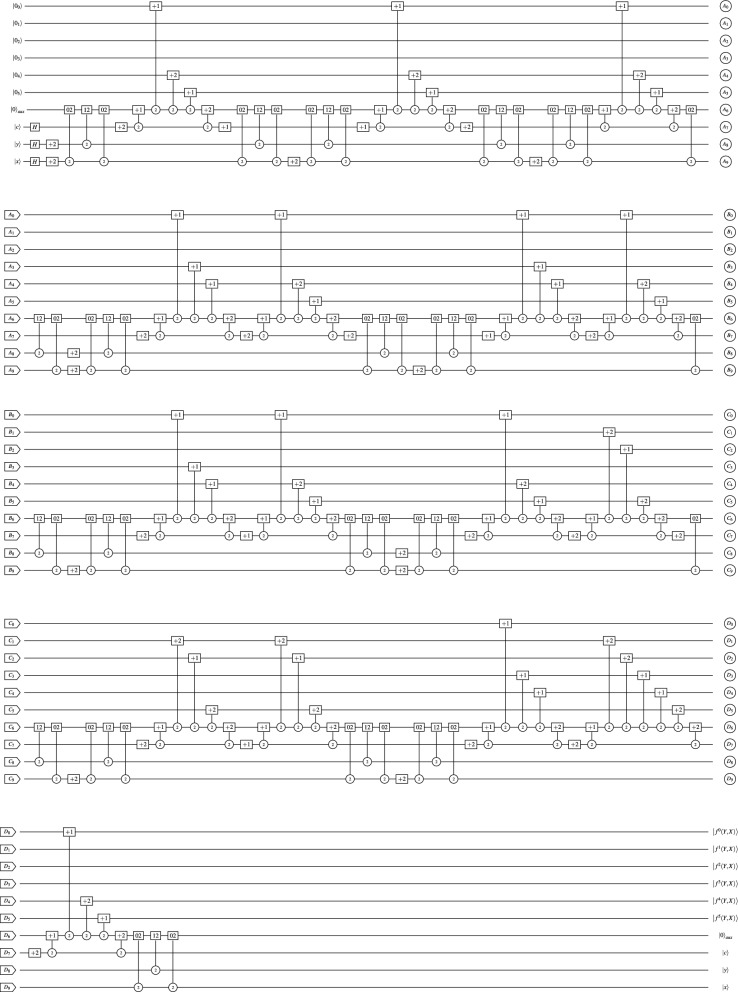
Phase II decomposition of the quantum circuit in Fig. 6, resulting in $QC = 170$.

This phase applies the movement, elimination, and merging rules described in^15^, as shown in Fig. 9. To demonstrate this phase on the TNCQR circuit shown in Fig. 8, the optimized version is presented in Fig. 10, resulting in a total quantum cost of $QC = 170$. This optimization phase eliminates or merges one-qutrit gates in the following 4 cases:

1. Between the open and close pixel position gates of the same pixel (red rectangles in Fig. 8a).
2. Between the open and close gates of the same color channel within a single pixel (green rectangles in Fig. 8a, 8b).
3. Between the close gate of one color and the open gate on the next color within the same pixel (purple rectangle in Fig. 8b).
4. Between the close pixel gate of one pixel and the open gate of the next pixel (blue rectangles in Fig. 8a).

### Phase III

This is the final phase, which is not as general as Phases I and II since it depends on the image and its grayscale or RGB color values. In this phase, each of the 6 color qutrits (*q*) is converted into a Boolean expression, simplified using Boolean algebra rules, and then reconstructed into a ternary circuit based on the simplified equations, as described in^15,17^.

The only case in which the TNCQR model can apply this phase is when a common factor appears, such as $Y = 1$ and $X = 0, 1, 2$, all sharing the same target *Z* gate. In this situation, the 3 corresponding three-qutrit gates can be replaced by 1 two-qutrit gate, where $Y = 1$ acts as the control and *Z* as the target, as demonstrated in grayscale example shown in Sect. “Performance analysis”. For RGB images, this condition requires that the common factor involves the same color channel, which is rarely achievable. Therefore, this phase does not appear in the current RGB image example.

## Performance analysis

This section presents a comparative analysis of the proposed TNCQR model against related previous models in both binary and ternary quantum systems, including the Enhanced NCQI (ENCQI) model^14^ for binary quantum representation, and the QTRQ^15^ and HQDQR^16^ models for ternary quantum representation. The comparison includes the total number of gates used to represent the image, quantum cost, and time complexity. All quantum circuits mentioned in this paper were implemented using the Cirq simulator^39^, which supports the simulation of multi-valued quantum systems. Since the original QTRQ model^15^ did not provide explicit quantum cost values, the results obtained for the QTRQ in this section were calculated manually to enable a fair comparison.

To compare the total number of gates used to represent the RGB digital image for each model, the TNCQR model uses $2n+1+ 23 \times 3^{2n}$ gates to represent the entire RGB digital image of size $3^n \times 3^n$. The term $2n+1$ represents the number of *H* gates used on $2n+1$ qutrits to get all pixel positions and the three color channels. The term $3^{2n}$ is the number of pixels, with 21 two-qutrit gates used to set the color value for each pixel and 2 *N*-qutrit gates to detect the pixel position.

QTRQ model uses $2n+1+ 15 \times 3^{2n}$ gates to represent $3^{2n}$ pixels, using 15 *N*-qutrit gates for each pixel in addition to $2n+1$ *H* gates. The HQDQR model uses also 15 *N*-qutrit gates to set the color value for each pixel in a rectangular image of size $3^m \times 2^n$, in addition to $m+n+1$ *H* gates. The ENCQI model uses 24 two-qubit gates to set the color value of one pixel, 2 2*n*-CNOT gates to detect the pixel position, and 2*n* *H* gates. Hence, it uses $2n + 26 \times 2^{2n}$ gates to represent a digital colored image of size $2^n \times 2^n$.

Table 1 shows the total number of gates used in each representation model relative to image size *n*, where *q* is the number of qudits required to represent the RGB color value for every pixel in each model. This comparison shows that the TNCQR model uses more two-qutrit gates than other models that use *N*-qutrit gates, which leads to decrease the cost in TNCQR model.

**Table 1:** Comparison between TNCQR model and related previous models in terms of *q*, total number of qudits, gate count and time complexity for each model.

Model	Image size	q	Number of qudits	Gate count	Time complexity
ENCQI^14^	$2^n\times 2^n$	24 qubits	$2n+q+1$	$2n+2^{2n}\times 26$	$O(2n2^{2n})$
QTRQ (RGB)^15^	$3^n\times 3^n$	7 qutrits	$2n+q+1$	$(2n+1)+3^{2n}\times 15$	$O(qn3^{2n})$
HQDQR^16^	$2^n\times 3^m$	7 qutrits	$(m+n)+q+1$	$(m+n+1)+(3^m\times 2^n)\times 15$	$O((n+m)\times 2^n\times 3^m)$
TNCQR	$3^n\times 3^n$	7 qutrits	$2n+q+2$	$(2n+1)+3^{2n}\times 23$	$O(n3^{2n})$

To compare the time complexity of each representation model, the TNCQR model has a time complexity of $O(n3^{2n})$ for representing $3^n \times 3^n$ colored images, where *n* determines the image size and 2*n* is the number of qutrits used to store position information of image pixels. The QTRQ model has a time complexity of $O(qn3^{2n})$ for representing $3^n \times 3^n$ colored images with a color range of $3^q$ and $q=6$ qutrits. The hybrid HQDQR model has a time complexity of $O((m+n) \times 2^n \times 3^m)$ for representing rectangular colored images of size $2^n \times 3^m$, where *n* is the number of qubits and *m* is the number of qutrits used to represent the size of the rectangular image. The ENCQI model is a binary quantum model that uses only qubits to represent $2^n \times 2^n$ colored images with a time complexity of $O(2n2^{2n})$, as shown in Table 1.

The comparison in terms of quantum cost is presented in a general form that represents the maximum *QC* for images of any size (worst-case scenario, where 5 qutrit values are changed per color channel for each pixel), as shown in Table 2. The comparison includes the proposed TNCQR model and the QTRQ model for RGB image representation, in terms of the total number of qutrits *M* used and the total quantum cost (*QC*) for different image sizes. The total QC of the proposed TNCQR model is calculated using the formula $QC_{t}=2n+1+\sum _{Y=0}^{3^n-1}\sum _{X=0}^{3^n-1}2\times (YX)_{QC}+29$, where $2n+1$ is the QC for the $2n+1$ *H* gates and $(YX)_{QC}$ represents the QC of the generalized *N*-qutrit gate, which corresponds to the pixel at position (*Y*, *X*). $(YX)_{QC}=4N+2k-7$ where $N=2n+1$ for $3^n\times 3^n$ image size and *k* is the number of controls not in state $|2\rangle$.

The total QC of the QTRQ model^15^ for RGB digital images is given by $QC_{t}=2n+1+\sum _{Y=0}^{3^n-1}\sum _{X=0}^{3^n-1}5\times (YX)_{R}+5\times (YX)_{G}+5\times (YX)_{B}$, where $2n+1$ is the QC for the $2n+1$ *H* gates and $(YX)_{R}$, $(YX)_{G}$ and $(YX)_{B}$ represent the QC of the generalized *N*-qutrit gate corresponding to one pixel at position (*Y*, *X*) for the colors Red, Green and Blue respectively. The QC for each color in one pixel $(YX)_{R}$, $(YX)_{G}$ or $(YX)_{B}$ is $4N+2k-7$, where $N=2n+2$ for $3^n\times 3^n$ image size and *k* is the number of controls not in state $|2\rangle$. When comparing the QC of the TNCQR model with the QC of the QTRQ model, it is found that the proposed model has a lower QC.

The total number of qutrits *M* used in TNCQR is $M=2n+q+2$ qutrits, where $q=6$ is fixed and represents the number of qutrits used to represent each color range. The number of qutrits *M* used in the TNCQR model is greater than in the QTRQ model by 1. However, the total QC for each image size in TNCQR is much lower than in the QTRQ model, as shown in Table 2, due to the ancilla qutrit which loads the position and decreases the number of generalized *N*-qutrit gates, replacing them with 21 two-qutrit gates to set the color value for each pixel.

**Table 2:** Comparison between TNCQR model and QTRQ model in terms of total number of qutrits *M* used in each model, where $q=6$, and the total QC. The total QC for the QTRQ model is $QC_{t}^{^1}=2n+1+ \sum _{Y=0}^{3^n-1}\sum _{X=0}^{3^n-1}5 \times (YX)_{R} + 5 \times (YX)_{G} + 5 \times (YX)_{B}$ and the total QC for TNCQR model is $QC_{t}^{^2}=2n+1+ \sum _{Y=0}^{3^n-1}\sum _{X=0}^{3^n-1}2\times (YX)_{QC}+29$ for representing $3^n\times 3^n$ RGB images of size *n*.

Image Size		$QTRQ_{_{RGB}}$		*TNCQR*	
$\quad n$	$3^n\times 3^n$	$M=2n+q+1$	$QC_{t}^{^1}$	$M=2n+q+2$	$QC_{t}^{^2}$
1	$3\times 3$	9	1,758	10	402
2	$9\times 9$	11	28,760	12	5,324
3	$27\times 27$	13	375,442	14	63,430
4	$81\times 81$	15	4,428,684	16	710,784
5	$243\times 243$	17	49,305,926	18	7,656,698
6	$729\times 729$	19	528,783,808	20	80,247,604
7	$2187\times 2187$	21	5,524,329,210	22	824,265,006

These comparisons prove the efficiency of the proposed TNCQR model over recent relevant models in terms of QC and time complexity. For example, encoding the RGB image in Fig. 5 using the TNCQR model produces a quantum circuit with 10 qutrits and an initial quantum cost of $QC = 267$, which is reduced to $QC = 170$ after optimization. In contrast, implementing the same image using 9 qutrits in both the QTRQ and HQDQR models results in a quantum cost of $QC = 637$. Therefore, regardless of the total number of gates used to represent an RGB image in a ternary quantum system, the TNCQR model uses fewer elementary gates (one and two-qutrit gates) than previous models. This leads to a more efficient representation of colored image in terms of lower cost and time complexity.

In the meantime, the proposed TNCQR model is applicable to grayscale images. Figure 1 presents a $3 \times 3$ grayscale image with 9 pixels and grayscale values in the range of 0 to 255, to be represented using the TNCQR model. To represent the 9 pixels for a $3 \times 3$ gray image, $q+1+2n$ qutrits are required. Setting *n* to 1 and *q* to 6 results in a 9 qutrits required to represent the 9 pixels positions in the $3 \times 3$ gray image. To construct the quantum circuit for the example image in Fig. 1 using the TNCQR model, begin by applying 2*H* gates to the 2 qutrits that define the pixel positions. Then, use a three-qutrit gate to load one pixel position onto the ancilla qutrit. Next, use up to 5 two-qutrit gates to set the gray value for this pixel. Once the gray value is set, a three-qutrit gate closes the pixel’s position. This process is repeated for all pixels in the image. Representing a grayscale image using the TNCQR model involves using a $[+2]$ gate on the target qutrit for the open position gates and a $[+1]$ gate on the target qutrit for the close position gates.

**Fig. 11:**
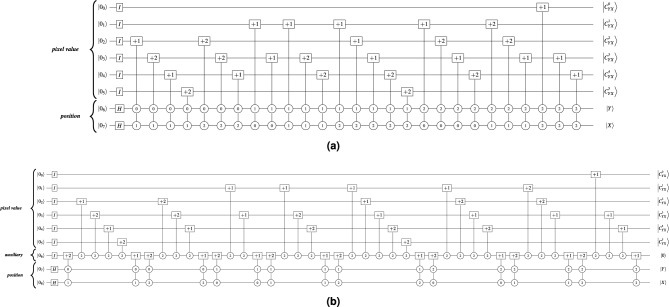
Ternary quantum circuit that represent the implementation of a $3\times 3$ grayscale example image shown in Fig. 1 using: (**a**) The QTRQ model with $QC = 203$^15^. (**b**) The proposed TNCQR model with $QC=149$.

The quantum circuit representing the $3 \times 3$ grayscale image using the TNCQR model is shown in Fig. 11b, with an initial quantum cost of $QC = 149$ and a reduced cost of $QC = 85$ after applying the three optimization phases. In comparison, Fig. 11a presents the quantum circuit for the same grayscale image using the QTRQ model, which achieves $QC = 203$ initially and $QC = 107$ after optimization. The model proposed in^17^ reaches a final quantum cost of $QC = 85$ after optimization.

The TNCQR circuit in Fig. 11b is then optimized through the 3 phases in Sect. “TNCQR Optimization algorithm”. In phase III The Boolean algebraic expression for the q qutrits from $|C^{^0}_{_{YX}}\rangle$ to $|C^{^5}_{_{YX}}\rangle$ are shown in Eq. 17. In these equation the common condition is achieved in $|C^{^1}_{_{YX}}\rangle$ where $Y=1$, and $X=0,1,2$ and $Z=+1$, and in $|C^{^3}_{_{YX}}\rangle$ where $Y=2$, and $X=0,1,2$ and $Z=+1$. In each case the 3 two-qutrit gates are replaces by 1 two-qutrit gate with *Y* control and *Z* target.

17 $$\begin{aligned} \begin{aligned} |C^{^0}_{_{YX}}\rangle&=Z(+1)Y^2X^2\\ |C^{^1}_{_{YX}}\rangle&=Z(+1)Y^1X^0+Z(+1)Y^1X^1+Z(+1)Y^1X^2+Z(+1)Y^2X^0+Z(+2)Y^2X^1\\&=Z(+1)Y^1+Z(+1)Y^2X^0+Z(+2)Y^2X^1\\ |C^{^2}_{_{YX}}\rangle&=Z(+1)Y^0X^1+Z(+1)Y^1X^2+Z(+2)Y^0X^2+Z(+2)Y^2X^0+Z(+2)Y^2X^1\\ |C^{^3}_{_{YX}}\rangle&=Z(+1)Y^1X^0+Z(+1)Y^1X^2+Z(+1)Y^2X^0+Z(+1)Y^2X^1+Z(+1)Y^2X^2+Z(+2)Y^0X^1+Z(+2)Y^0X^2+Z(+2)Y^1X^1\\&=Z(+1)Y^1X^0+Z(+1)Y^1X^2+Z(+1)Y^2+Z(+2)Y^0X^1+Z(+2)Y^0X^2+Z(+2)Y^1X^1\\ |C^{^4}_{_{YX}}\rangle&=Z(+1)Y^0X^1+Z(+1)Y^0X^2+Z(+1)Y^2X^2+Z(+2)Y^1X^1+Z(+2)Y^1X^2+Z(+2)Y^2X^0+Z(+2)Y^0X^2+Z(+2)Y^1X^1\\ |C^{^5}_{_{YX}}\rangle&=Z(+1)Y^0X^1+Z(+1)Y^1X^2 \end{aligned} \end{aligned}$$

Table 3 presents a comparison between the TNCQR and QTRQ models in terms of QC under the worst-case scenario for $3^n\times 3^n$ grayscale images with different sizes *n*. The QC for the TNCQR model is calculated by $2n+\sum _{Y=0}^{3^n-1}\sum _{X=0}^{3^n-1}2\times (YX)_{QC}+5$, while the QC for the QTRQ model is calculated by $2n+\sum _{Y=0}^{3^n-1}\sum _{X=0}^{3^n-1} 5 \times (YX)_{QC}$. The 2*n* term is the QC for the 2*n* *H* gates and $(YX)_{QC}$ represents the QC of the generalized *N*-qutrit gate, which corresponds to the pixel at position (*Y*, *X*). $(YX)_{QC}=4N+2k-7$ for both models where $N=2n+1$ for $3^n\times 3^n$ image size and *k* is the number of controls not in state $|2\rangle$. The comparison shows the efficiency of the proposed TNCQR model than the QTRQ model for representing grayscale images in ternary quantum system.

**Table 3:** Comparison between TNCQR model and QTRQ model for grayscale images in terms of total number of qutrits *M* used in each model, where $q=6$, the total QC for QTRQ model is $QC_{t}^{^1}=2n+\sum _{Y=0}^{3^n-1}\sum _{X=0}^{3^n-1}5\times (YX)_{QC}$ and the total QC for TNCQR model is $QC_{t}^{^2}=2n+\sum _{Y=0}^{3^n-1}\sum _{X=0}^{3^n-1}2\times (YX)_{QC}+5$ for different $3^n\times 3^n$ images sizes *n*.

Image size		$QTRQ_{Gray}$		$TNCQR_{Gray}$	
$\quad n$	$3^n\times 3^n$	$M=2n+q$	$QC_{t}^{^1}$	$M=2n+q+1$	$QC_{t}^{^2}$
1	$3\times 3$	8	347	9	185
2	$9\times 9$	10	7,429	11	3,379
3	$27\times 27$	12	105,711	13	45,933
4	$81\times 81$	14	1,301,273	15	553,319
5	$243\times 243$	16	14,860,675	17	6,239,521
6	$729\times 729$	18	162,089,517	19	67,493,019
7	$2187\times 2187$	20	1,713,897,239	21	709,473,749

## Conclusion

Quantum image processing (QIP) using qutrits offers more efficient image processing technologies by leveraging the unique properties of ternary quantum systems. The expanded state space of qutrits provides significant advantages in terms of data representation, computational efficiency, and algorithmic flexibility.

This paper proposes a novel qutrit-based model, the Ternary Novel Colored Quantum Representation (TNCQR), for encoding $3^n \times 3^n$ RGB digital images on a ternary quantum system. The TNCQR model efficiently encodes RGB color values (ranging from 0 to 255) using only 7 qutrits per pixel and requires fewer elementary gates (one-qutrit and two-qutrit gates) compared to existing models, resulting in lower quantum cost and reduced time complexity. The time complexity of the TNCQR model is $O(n3^{2n})$, and its overall quantum cost (*QC*) is lower than the related models in the literature. Comparative analysis with QTRQ, HQDQR, and ENEQR confirms that TNCQR achieves better performance in terms of the number of elementary gates, quantum cost, and time complexity for RGB image representation on ternary quantum systems. Moreover, the model can also encode grayscale images while maintaining a lower quantum cost than the ternary grayscale QTRQ model.

An optimization algorithm comprising 3 phases is introduced to minimize the quantum cost of circuits constructed by the TNCQR model. For example, applying this optimization to the $3 \times 3$ RGB image shown in Fig. 5 reduces the quantum cost from $QC = 267$ to $QC = 170$, achieving a $36\%$ reduction. These improvements establish TNCQR as an efficient, scalable, and resource-optimized framework for quantum image representation in ternary systems.

This paper focuses on the algorithmic formulation and simulation-based validation of the proposed qutrit image encoding framework. Since commercial qutrit hardware is not yet available, the study establishes a foundation for future work aimed at mapping the TNCQR framework onto physical qutrit platforms once stable multi-level architectures become accessible. Finally, the TNCQR model demonstrates efficient image encoding and provides a basis for future implementations of advanced qutrit-based image processing operations such as filtering, transforms, and encryption. As quantum technology matures, the integration of qutrit systems into image processing workflows is expected to unlock new capabilities and accelerate the practical realization of quantum-enhanced image technologies across scientific and industrial domains.

## Supplementary Information

Supplementary Information.

## Data Availability

All data generated or analyzed during this study are included in the manuscript.
