# Supplementary material for: A novel qutrit representation for RGB digital images
Source: Sci Rep. 2025 Dec 5;15:43256. doi: 10.1038/s41598-025-27910-0 (PMC12680611; doi:10.1038/s41598-025-27910-0)
Supplement: Supplementary file 1 — Supplementary Information. [file 41598_2025_27910_MOESM1_ESM.pdf]

Supplementary Information for
A Novel Qutrit Representation for RGB Digital Images

Mirna Rofail[1], **Rasha Montaser**[2], **Ahmed Younes**[1,3]

[1]Department of Mathematics and Computer Science, Faculty of Science, Alexandria University, 21526, Egypt
[2]Department of Information Systems, Faculty of Computers and Information Science, Damanhour University, 22511, Egypt
[3]Faculty of Computer Science and Engineering, Alamein International University, 51718, Egypt

S1 Basic Definitions

In this section, the key definitions used throughout the supplementary are presented.

Definition 1 (Ancilla Qutrit). *An* ancilla *is a temporary, auxiliary three-level quantum system (qutrit) initialized in a specific known state, typically the* $|0\rangle$ *state*[1–6].

Definition 2 (Quantum Cost (*QC*)). *Quantum cost is a metric that assigns costs to gates according to the ternary quantum system architecture. Quantum cost evaluates complex (N-qutrit) gates by decomposing them into elementary ternary gates (one-qutrit and two-qutrit gates) and summing their costs*[1, 5, 7–9].

S2 Basics of Ternary Quantum Logic

The ternary quantum system has a unit of data representation called qutrit (quantum ternary digit) where it consists of three quantum base states $|0\rangle, |1\rangle$, and $|2\rangle$. Each state is represented by 3×1 vector, as shown in Eq (S1)[4,5,7],

$$|0\rangle = \begin{bmatrix}1\\0\\0\end{bmatrix}, \qquad |1\rangle = \begin{bmatrix}0\\1\\0\end{bmatrix}, \qquad |2\rangle = \begin{bmatrix}0\\0\\1\end{bmatrix}. \tag{S1}$$

Any ternary quantum state $|\psi\rangle$ is represented using the Dirac notation as Eq (S2), where α, β, γ are complex numbers that represent the amplitude of states $|0\rangle, |1\rangle, |2\rangle$, respectively. When applying the measurement, the probability of finding the state $|0\rangle$, $|1\rangle$, or $|2\rangle$ is $|\alpha|^2$, $|\beta|^2$, or $|\gamma|^2$, respectively. These probabilities must satisfy the normalization condition $|\alpha|^2 + |\beta|^2 + |\gamma|^2 = 1$[4,5,7].

$$|\psi\rangle = \alpha\,|0\rangle + \beta\,|1\rangle + \gamma|2\rangle, \tag{S2}$$

S2.1 One-qutrit gates (Ternary Shift Gates)

Ternary shift gates are unitary quantum gates that operate on a single qutrit to modify its state. There are six ternary shift gates (Z gates): [+0], [+1], [+2], [01], [02], [12]. Each of these gates can be represented by a 3×3 matrix as follows:[4–10]

$$[+0] = \begin{bmatrix}1&0&0\\0&1&0\\0&0&1\end{bmatrix}, \quad [+1] = \begin{bmatrix}0&0&1\\1&0&0\\0&1&0\end{bmatrix}, \quad [+2] = \begin{bmatrix}0&1&0\\0&0&1\\1&0&0\end{bmatrix},$$
$$[01] = \begin{bmatrix}0&1&0\\1&0&0\\0&0&1\end{bmatrix}, \quad [02] = \begin{bmatrix}0&0&1\\0&1&0\\1&0&0\end{bmatrix}, \quad [12] = \begin{bmatrix}1&0&0\\0&0&1\\0&1&0\end{bmatrix}. \tag{S3}$$

The $[+0]$ gate is similar to the identity gate (I). The $[+1]$ gate is a single-shift gate that transforms the state of a qutrit by adding 1 mod 3. The $[+2]$ gate is a double-shift gate that transforms the state of a qutrit by adding 2 mod 3. The [01] gate is a self-single-shift gate that swaps the state $|0\rangle$ with $|1\rangle$ of a single qutrit. The [02] gate is a self-double-shift gate that swaps the ternary state $|0\rangle$ with $|2\rangle$ of a single qutrit. The [12] gate is a self-shift gate that swaps the ternary state $|1\rangle$ with $|2\rangle$ of a single qutrit. Each of these gates has $QC = 1$[1,5,7–9].

Another important one-qutrit gate is the ternary Hadamard gate (H), which is responsible for the preparation of ternary superposition states. It can be represented by a 3×3 matrix as shown in Eq (S4)[6,7,11],

$$H = \frac{1}{\sqrt{3}} \begin{bmatrix} 1 & 1 & 1 \\ 1 & e^{\frac{2\pi i}{3}} & e^{\frac{4\pi i}{3}} \\ 1 & e^{\frac{4\pi i}{3}} & e^{\frac{8\pi i}{3}} \end{bmatrix}. \tag{S4}$$

When the H gate acts on an arbitrary ternary quantum state $|\psi\rangle$, as in Eq (S2), it produces the general form of the ternary superposition states, as shown in Eq (S5),

$$H|\psi\rangle = \frac{1}{\sqrt{3}}(\alpha+\beta+\gamma)|0\rangle + \frac{1}{\sqrt{3}}(\alpha+\beta e^{\frac{2\pi i}{3}}+\gamma e^{\frac{4\pi i}{3}})|1\rangle + \frac{1}{\sqrt{3}}(\alpha+\beta e^{\frac{4\pi i}{3}}+\gamma e^{\frac{8\pi i}{3}})|2\rangle. \tag{S5}$$

Figure S1 shows the symbolic notation for one-qutrit and H gates in quantum circuit, each with $QC = 1$. Table S1 presents the truth table for the seven one-qutrit gates: the six ternary shift gates and the ternary H gate.

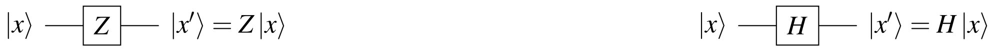

(a) (b)

Figure S1. The symbolic notation of one-qutrit gates in quantum circuit: (a) The notation of any Z gate. (b) The notation of ternary H gate.

Input	[+0]	[+1]	[+2]	[01]	[02]	[12]	H
$\|0\rangle$	$\|0\rangle$	$\|1\rangle$	$\|2\rangle$	$\|1\rangle$	$\|2\rangle$	$\|0\rangle$	$\frac{1}{\sqrt{3}}(\|0\rangle+\|1\rangle+\|2\rangle)$
$\|1\rangle$	$\|1\rangle$	$\|2\rangle$	$\|0\rangle$	$\|0\rangle$	$\|1\rangle$	$\|2\rangle$	$\frac{1}{\sqrt{3}}(\|0\rangle+e^{\frac{2\pi i}{3}}\|1\rangle+e^{\frac{4\pi i}{3}}\|2\rangle)$
$\|2\rangle$	$\|2\rangle$	$\|0\rangle$	$\|1\rangle$	$\|2\rangle$	$\|0\rangle$	$\|1\rangle$	$\frac{1}{\sqrt{3}}(\|0\rangle+e^{\frac{4\pi i}{3}}\|1\rangle+e^{\frac{8\pi i}{3}}\|2\rangle)$

Table S1. The Truth table for each one-qutrit gate[5,9,11].

Each one-qutrit gate has a unitary inverse gate to reverse its function to maintain reversibility in ternary quantum circuits. The $[+1]$ gate is the inverse of the $[+2]$ gate and vice versa. Each swap gate [01], [02], or [12] is its own inverse, meaning that applying the same gate twice cancels out its function. The same principle applies to the H gate[5,7,9].

S2.2 Two-qutrit gates (M-S gates)

Muthukrishnan and Stroud[12] proposed a family of two-qutrit multi-valued gates implemented using linear ion trap for quantum computing. The two-qutrit M-S gates are unitary quantum gates that operate on two qutrits: a control qutrit and a target qutrit. The gate on the target qutrit is applied when the control qutrit is in state $|2\rangle$ with $QC = 1$ as shown in Figure S2a[4–9,12,13]. The control qutrit can also be in state $|0\rangle$, or $|1\rangle$, as shown in Figure S2b. However, using $|0\rangle$ or $|1\rangle$ as the control state increases the gate cost by 2, as shown in the decomposition gate in Figure S2c. In this figure, $[+(2-a)]$ and $[+(1+a)])$ represent the gates $[+((2-a)\,mod\,3)]$ and $[+((1+a)\,mod\,3)]$, respectively, where $a \in \{|0\rangle, |1\rangle, |2\rangle\}$. A general formula to calculate QC of two-qutrit gates is $2k+1$, where k is the number of controls not in state $|2\rangle$[1,5,6,9,13].

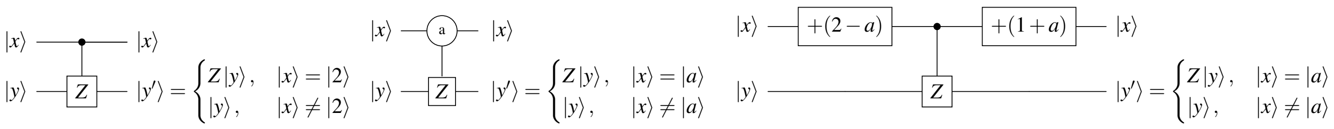

(a) (b) (c)

Figure S2. Symbolic notations for the two-qutrit gates in quantum circuit: (a) The default notation of two-qutrit gate when the control qutrit is in state $|2\rangle$. (b) The notation of generalized two-qutrit gate when the control qutrit $|x\rangle$ is in state $|a\rangle \in \{|0\rangle, |1\rangle, |2\rangle\}$. (c) The decomposition of the generalized two-qutrit gate.

Figure S3 presents the 6 possible two-qutrit gates in ternary quantum circuit, and Table S2 provides their corresponding truth table[1,4].

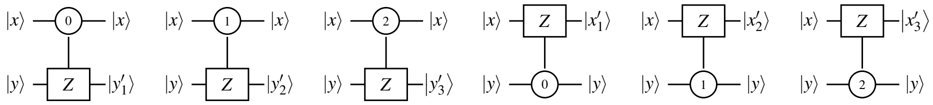

Figure S3. The 6 possible two-qutrit M-S gates in ternary quantum circuit.

$\lvert x\rangle$	$\lvert y\rangle$	$\lvert y'_1\rangle$	$\lvert y'_2\rangle$	$\lvert y'_3\rangle$	$\lvert x'_1\rangle$	$\lvert x'_2\rangle$	$\lvert x'_3\rangle$
$\lvert 0\rangle$	$\lvert 0\rangle$	$Z\lvert 0\rangle$	$\lvert 0\rangle$	$\lvert 0\rangle$	$Z\lvert 0\rangle$	$\lvert 0\rangle$	$\lvert 0\rangle$
$\lvert 0\rangle$	$\lvert 1\rangle$	$Z\lvert 1\rangle$	$\lvert 1\rangle$	$\lvert 1\rangle$	$\lvert 0\rangle$	$Z\lvert 0\rangle$	$\lvert 0\rangle$
$\lvert 0\rangle$	$\lvert 2\rangle$	$Z\lvert 2\rangle$	$\lvert 2\rangle$	$\lvert 2\rangle$	$\lvert 0\rangle$	$\lvert 0\rangle$	$Z\lvert 0\rangle$
$\lvert 1\rangle$	$\lvert 0\rangle$	$\lvert 0\rangle$	$Z\lvert 0\rangle$	$\lvert 0\rangle$	$Z\lvert 1\rangle$	$\lvert 1\rangle$	$\lvert 1\rangle$
$\lvert 1\rangle$	$\lvert 1\rangle$	$\lvert 1\rangle$	$Z\lvert 1\rangle$	$\lvert 1\rangle$	$\lvert 1\rangle$	$Z\lvert 1\rangle$	$\lvert 1\rangle$
$\lvert 1\rangle$	$\lvert 2\rangle$	$\lvert 2\rangle$	$Z\lvert 2\rangle$	$\lvert 2\rangle$	$\lvert 1\rangle$	$\lvert 1\rangle$	$Z\lvert 1\rangle$
$\lvert 2\rangle$	$\lvert 0\rangle$	$\lvert 0\rangle$	$\lvert 0\rangle$	$Z\lvert 0\rangle$	$Z\lvert 2\rangle$	$\lvert 2\rangle$	$\lvert 2\rangle$
$\lvert 2\rangle$	$\lvert 1\rangle$	$\lvert 1\rangle$	$\lvert 1\rangle$	$Z\lvert 1\rangle$	$\lvert 2\rangle$	$Z\lvert 2\rangle$	$\lvert 2\rangle$
$\lvert 2\rangle$	$\lvert 2\rangle$	$\lvert 2\rangle$	$\lvert 2\rangle$	$Z\lvert 2\rangle$	$\lvert 2\rangle$	$\lvert 2\rangle$	$Z\lvert 2\rangle$

Table S2. The Truth table for the 6 possible two-qutrit M-S gates shown in Figure S3[4].

S2.3 Three-qutrit M-S gates (Ternary Toffoli)

Khan and Perkowski[1] presented a three-qutrit generalized Toffoli gate along with its realization in ion-trap technology. The three-qutrit gates are unitary quantum gates that operate on three qutrits: two control qutrits and one target qutrit. The gate on the target qutrit is applied when both control qutrits are in state $\lvert 2\rangle$, as shown in Figure S4a. The QC for three-qutrit M-S gate is 5, because a three-qutrit gate is decomposed into 5 two-qutrit gates, as shown in Figure S4b[1,4–7,9].

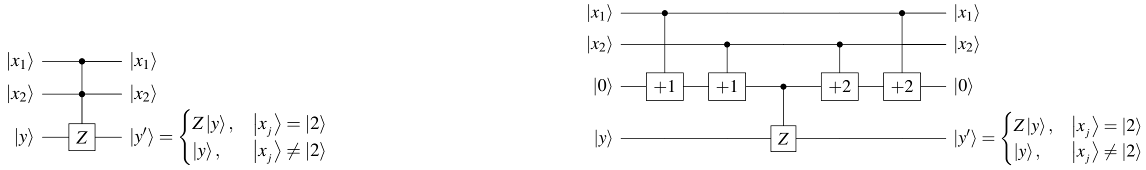

(a) (b)

Figure S4. The symbolic notation for three-qutrit gates in quantum circuit: (a) The default notation of three-qutrit gate when both control qutrits $\lvert x_j\rangle$ are in state $\lvert 2\rangle$, where $j \in \{1,2\}$. (b) The decomposition of default three-qutrit gate into 5 two-qutrit M-S gates.

Control qutrits can operate on any qutrit state $\lvert 0\rangle, \lvert 1\rangle$ or $\lvert 2\rangle$ which generates a generalized three-qutrit gate, as shown in Figure S5a. Figure S5b shows the decomposition of a generalized three-qutrit gate. The control state affects the gate cost, resulting in the general formula to calculate the QC of a generalized three-qutrit gate as $QC = 2k+5$, where k is the number of controls not in state $\lvert 2\rangle$[1,5,9].

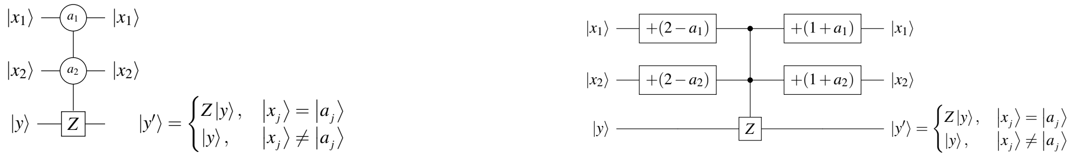

(a) (b)

Figure S5. The symbolic notation for generalized three-qutrit gate in quantum circuit: (a) A three-qutrit gate with the control qutrits $\lvert x_j\rangle$ in any state $\lvert a_j\rangle \in \{\lvert 0\rangle, \lvert 1\rangle, \lvert 2\rangle\}$, where $j \in \{1,2\}$ (b) The decomposition of generalized three-qutrit gate.

Figure S6 shows 9 out of the 27 possible three-qutrit gates in ternary quantum circuit, and Table S3 provides their corresponding truth table[1,4].

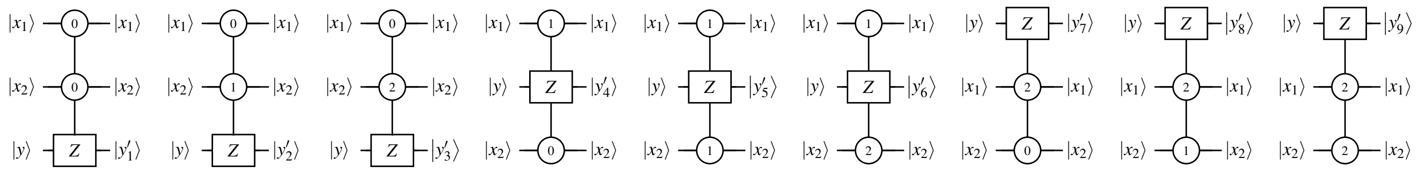

Figure S6. 9 out of the 27 possible three-qutrit M-S gates in ternary quantum circuit.

$\|x_1\rangle$	$\|x_2\rangle$	$\|y\rangle$	$\|y'_1\rangle$	$\|y'_2\rangle$	$\|y'_3\rangle$	$\|y'_4\rangle$	$\|y'_5\rangle$	$\|y'_6\rangle$	$\|y'_7\rangle$	$\|y'_8\rangle$	$\|y'_9\rangle$
$\|0\rangle$	$\|0\rangle$	$\|0\rangle$	$Z\|0\rangle$	$\|0\rangle$	$\|0\rangle$	$\|0\rangle$	$\|0\rangle$	$\|0\rangle$	$\|0\rangle$	$\|0\rangle$	$\|0\rangle$
$\|0\rangle$	$\|0\rangle$	$\|1\rangle$	$Z\|1\rangle$	$\|1\rangle$	$\|1\rangle$	$\|1\rangle$	$\|1\rangle$	$\|1\rangle$	$\|1\rangle$	$\|1\rangle$	$\|1\rangle$
$\|0\rangle$	$\|0\rangle$	$\|2\rangle$	$Z\|2\rangle$	$\|2\rangle$	$\|2\rangle$	$\|2\rangle$	$\|2\rangle$	$\|2\rangle$	$\|2\rangle$	$\|2\rangle$	$\|2\rangle$
$\|0\rangle$	$\|1\rangle$	$\|0\rangle$	$\|0\rangle$	$Z\|0\rangle$	$\|0\rangle$	$\|0\rangle$	$\|0\rangle$	$\|0\rangle$	$\|0\rangle$	$\|0\rangle$	$\|0\rangle$
$\|0\rangle$	$\|1\rangle$	$\|1\rangle$	$\|1\rangle$	$Z\|1\rangle$	$\|1\rangle$	$\|1\rangle$	$\|1\rangle$	$\|1\rangle$	$\|1\rangle$	$\|1\rangle$	$\|1\rangle$
$\|0\rangle$	$\|1\rangle$	$\|2\rangle$	$\|2\rangle$	$Z\|2\rangle$	$\|2\rangle$	$\|2\rangle$	$\|2\rangle$	$\|2\rangle$	$\|2\rangle$	$\|2\rangle$	$\|2\rangle$
$\|0\rangle$	$\|2\rangle$	$\|0\rangle$	$\|0\rangle$	$\|0\rangle$	$Z\|0\rangle$	$\|0\rangle$	$\|0\rangle$	$\|0\rangle$	$\|0\rangle$	$\|0\rangle$	$\|0\rangle$
$\|0\rangle$	$\|2\rangle$	$\|1\rangle$	$\|1\rangle$	$\|1\rangle$	$Z\|1\rangle$	$\|1\rangle$	$\|1\rangle$	$\|1\rangle$	$\|1\rangle$	$\|1\rangle$	$\|1\rangle$
$\|0\rangle$	$\|2\rangle$	$\|2\rangle$	$\|2\rangle$	$\|2\rangle$	$Z\|2\rangle$	$\|2\rangle$	$\|2\rangle$	$\|2\rangle$	$\|2\rangle$	$\|2\rangle$	$\|2\rangle$
$\|1\rangle$	$\|0\rangle$	$\|0\rangle$	$\|0\rangle$	$\|0\rangle$	$\|0\rangle$	$Z\|0\rangle$	$\|0\rangle$	$\|0\rangle$	$\|0\rangle$	$\|0\rangle$	$\|0\rangle$
$\|1\rangle$	$\|0\rangle$	$\|1\rangle$	$\|1\rangle$	$\|1\rangle$	$\|1\rangle$	$Z\|1\rangle$	$\|1\rangle$	$\|1\rangle$	$\|1\rangle$	$\|1\rangle$	$\|1\rangle$
$\|1\rangle$	$\|0\rangle$	$\|2\rangle$	$\|2\rangle$	$\|2\rangle$	$\|2\rangle$	$Z\|2\rangle$	$\|2\rangle$	$\|2\rangle$	$\|2\rangle$	$\|2\rangle$	$\|2\rangle$
$\|1\rangle$	$\|1\rangle$	$\|0\rangle$	$\|0\rangle$	$\|0\rangle$	$\|0\rangle$	$\|0\rangle$	$Z\|0\rangle$	$\|0\rangle$	$\|0\rangle$	$\|0\rangle$	$\|0\rangle$
$\|1\rangle$	$\|1\rangle$	$\|1\rangle$	$\|1\rangle$	$\|1\rangle$	$\|1\rangle$	$\|1\rangle$	$Z\|1\rangle$	$\|1\rangle$	$\|1\rangle$	$\|1\rangle$	$\|1\rangle$
$\|1\rangle$	$\|1\rangle$	$\|2\rangle$	$\|2\rangle$	$\|2\rangle$	$\|2\rangle$	$\|2\rangle$	$Z\|2\rangle$	$\|2\rangle$	$\|2\rangle$	$\|2\rangle$	$\|2\rangle$
$\|1\rangle$	$\|2\rangle$	$\|0\rangle$	$\|0\rangle$	$\|0\rangle$	$\|0\rangle$	$\|0\rangle$	$\|0\rangle$	$Z\|0\rangle$	$\|0\rangle$	$\|0\rangle$	$\|0\rangle$
$\|1\rangle$	$\|2\rangle$	$\|1\rangle$	$\|1\rangle$	$\|1\rangle$	$\|1\rangle$	$\|1\rangle$	$\|1\rangle$	$Z\|1\rangle$	$\|1\rangle$	$\|1\rangle$	$\|1\rangle$
$\|1\rangle$	$\|2\rangle$	$\|2\rangle$	$\|2\rangle$	$\|2\rangle$	$\|2\rangle$	$\|2\rangle$	$\|2\rangle$	$Z\|2\rangle$	$\|2\rangle$	$\|2\rangle$	$\|2\rangle$
$\|2\rangle$	$\|0\rangle$	$\|0\rangle$	$\|0\rangle$	$\|0\rangle$	$\|0\rangle$	$\|0\rangle$	$\|0\rangle$	$\|0\rangle$	$Z\|0\rangle$	$\|0\rangle$	$\|0\rangle$
$\|2\rangle$	$\|0\rangle$	$\|1\rangle$	$\|1\rangle$	$\|1\rangle$	$\|1\rangle$	$\|1\rangle$	$\|1\rangle$	$\|1\rangle$	$Z\|1\rangle$	$\|1\rangle$	$\|0\rangle$
$\|2\rangle$	$\|0\rangle$	$\|2\rangle$	$\|2\rangle$	$\|2\rangle$	$\|2\rangle$	$\|2\rangle$	$\|2\rangle$	$\|2\rangle$	$Z\|2\rangle$	$\|2\rangle$	$\|2\rangle$
$\|2\rangle$	$\|1\rangle$	$\|0\rangle$	$\|0\rangle$	$\|0\rangle$	$\|0\rangle$	$\|0\rangle$	$\|0\rangle$	$\|0\rangle$	$\|0\rangle$	$Z\|0\rangle$	$\|0\rangle$
$\|2\rangle$	$\|1\rangle$	$\|1\rangle$	$\|1\rangle$	$\|1\rangle$	$\|1\rangle$	$\|1\rangle$	$\|1\rangle$	$\|1\rangle$	$\|1\rangle$	$Z\|1\rangle$	$\|1\rangle$
$\|2\rangle$	$\|1\rangle$	$\|2\rangle$	$\|2\rangle$	$\|2\rangle$	$\|2\rangle$	$\|2\rangle$	$\|2\rangle$	$\|2\rangle$	$\|2\rangle$	$Z\|2\rangle$	$\|2\rangle$
$\|2\rangle$	$\|2\rangle$	$\|0\rangle$	$\|0\rangle$	$\|0\rangle$	$\|0\rangle$	$\|0\rangle$	$\|0\rangle$	$\|0\rangle$	$\|0\rangle$	$\|0\rangle$	$Z\|0\rangle$
$\|2\rangle$	$\|2\rangle$	$\|1\rangle$	$\|1\rangle$	$\|1\rangle$	$\|1\rangle$	$\|1\rangle$	$\|1\rangle$	$\|1\rangle$	$\|1\rangle$	$\|1\rangle$	$Z\|1\rangle$
$\|2\rangle$	$\|2\rangle$	$\|2\rangle$	$\|2\rangle$	$\|2\rangle$	$\|2\rangle$	$\|2\rangle$	$\|2\rangle$	$\|2\rangle$	$\|2\rangle$	$\|2\rangle$	$Z\|2\rangle$

Table S3. The Truth table for the 9 three-qutrit M-S gates shown in Figure S6[4].

S2.4 Generalized N-qutrit M-S gate (Generalized Ternary Toffoli)

To implement the unitary generalized N-qutrit M-S gate, the N qutrits are divided into $N-1$ control qutrits and one target qutrit. The Z gate on the target qutrit is activated when all $N-1$ control qutrits are in state $|2\rangle$, as shown in Figure S7a, with $QC = 4N-7$, where $N \geq 2$ qutrits[6].

To decompose the N-qutrit gate, first add $N-2$ ancilla qutrits in state $|0\rangle$, then the $N-1$ control qutrits are decomposed into $2N-4$ two-qutrit gates with a target $[+1]$ gate, followed by 1 two-qutrit gate with a target Z gate, and finally $2N-4$ two-qutrit gates with a target $[+2]$ gate. Figure S7b illustrates the decomposition of the N-qutrit M-S gate[1,6,8].

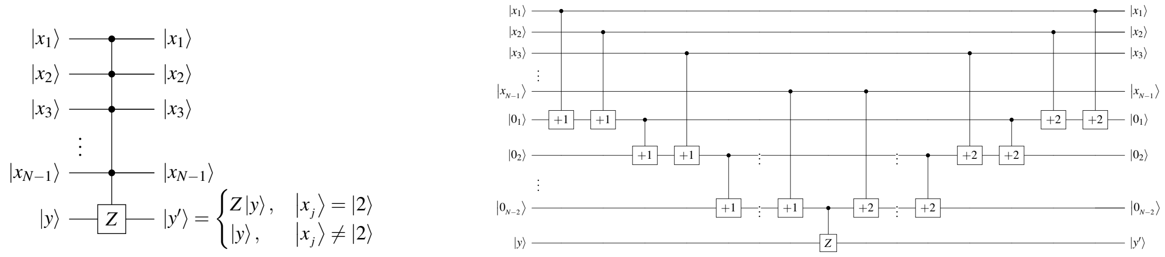

(a) **(b)**

Figure S7. Symbolic notation for the generalized N-qutrit gate in quantum circuit: (a) The default notation of N-qutrit gate with $N-1$ control qutrits $\left|x_j\right\rangle$ all in state $|2\rangle$, where $j \in \{1,2,3,\ldots,N-1\}$ (b) The decomposition of generalized N-qutrit gate.

While qutrit controls can operate in any state: $|0\rangle$, $|1\rangle$, or $|2\rangle$, an N-qutrit gate can operate with any control state, as shown in Figure S8a. Figure S8b illustrates the decomposition of an N-qutrit gate with any control state. The QC for this gate is calculated by $QC = 4N + 2k - 7$[6], where N is the number of qutrits used in the gate, and k is the number of controls not in state $|2\rangle$.

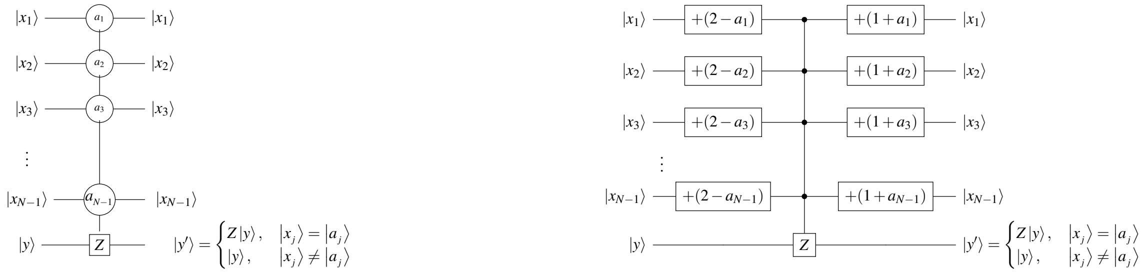

(a) **(b)**

Figure S8. Symbolic notation for the generalized N-qutrit gate in quantum circuit: (a) The generalized N-qutrit gate with control qutrits $\left|x_j\right\rangle$ function according to state $\left|a_j\right\rangle \in \{|0\rangle, |1\rangle, |2\rangle\}$, where $j \in \{1,2,3,\ldots,N-1\}$ (b) The decomposition of generalized N-qutrit gate.

References

1. Khan, M. H. & Perkowski, M. A. Quantum ternary parallel adder/subtractor with partially-look-ahead carry. *J. Syst. Archit.* **53**, 453–464, DOI: https://doi.org/10.1016/j.sysarc.2007.01.007 (2007).
2. Sang, J., Wang, S. & Li, Q. A novel quantum representation of color digital images. *Quantum Inf. Process.* **16**, 42, DOI: 10.1007/s11128-016-1463-0 (2016).
3. Bennakhi, A., Byrd, G. T. & Franzon, P. Analyzing quantum circuit depth reduction with ancilla qubits in mcx gates. In Culhane, C., Byrd, G., Muller, H., Alexev, Y. & Sheldon, S. (eds.) *2024 IEEE INTERNATIONAL CONFERENCE ON QUANTUM COMPUTING AND ENGINEERING, QCE, VOL 2*, 510–511, DOI: 10.1109/QCE60285.2024.10380. Microsoft; Keysight Technologies Inc; Quantinuum Ltd; Qunasys Inc; Psiquantum Corp (IEEE COMPUTER SOC, 10662 LOS VAQUEROS CIRCLE, PO BOX 3014, LOS ALAMITOS, CA 90720-1264 USA, 2024). 2024 International Conference on Quantum Computing and Engineering, Montreal, CANADA, SEP 15-20, 2024.
4. Rani, P. M. N. & Datta, K. Improved ternary reversible logic synthesis using group theoretic approach. *J. Circuits, Syst. Comput.* **29**, 2050192, DOI: 10.1142/S0218126620501923 (2020).
5. Monfared, A. T. & Haghparast, M. Quantum ternary multiplication gate (qtmg): Toward quantum ternary multiplier and a new realization for ternary toffoli gate. *J. Circuits, Syst. Comput.* **29**, 2050071, DOI: 10.1142/S0218126620500711 (2020).
6. Dong, H., Lu, D. & Li, C. A novel qutrit representation of quantum image. *Quantum Inf. Process.* **21**, 108, DOI: 10.1007/s11128-022-03450-8 (2022).
7. Taheri M., A., Ciriani, V. & Haghparast, M. Qutrit representation of quantum images: new quantum ternary circuit design. *Quantum Inf. Process.* **23**, 288, DOI: 10.1007/s11128-024-04484-w (2024).

8. Khan, M., Perkowski, M., Khan, M. & Kerntopf, P. Ternary gfsop minimization using kronecker decision diagrams and their synthesis with quantum cascades. *JOURNAL OF MULTIPLE-VALUED LOGIC AND SOFT COMPUTING* **11**, 567–602 (2005).

9. Khan, M. H. A. Design of reversible/quantum ternary multiplexer and demultiplexer. *Eng. Lett.* **13**, 65–69 (2006).

10. Das, S. & Caruso, F. A hybrid-qudit representation of digital rgb images. *Sci. Reports* **13**, 13671, DOI: 10.1038/s41598-023-39906-9 (2023).

11. Chen, G., Wang, Y., Jian, L., Zhou, Y. & Liu, S. Ternary quantum key distribution protocol based on hadamard gate. *Int. J. Theor. Phys.* **61**, 26, DOI: 10.1007/s10773-022-05041-w (2022).

12. Muthukrishnan, A. & Stroud, C. R. Multivalued logic gates for quantum computation. *Phys. Rev. A* **62**, 052309, DOI: 10.1103/PhysRevA.62.052309 (2000).

13. Fan, F., Yang, G., Yang, G. & Hung, W. N. N. A synthesis method of quantum reversible logic circuit based on elementary qutrit quantum logic gates. *J. Circuits, Syst. Comput.* **24**, 1550121, DOI: 10.1142/S0218126615501212 (2015).
